# LSD1-GLS2 axis drives subtype-specific chemoresistance in pancreatic cancer through glutaminolysis reprogramming

**DOI:** 10.1038/s41419-026-09075-4

**Published:** 2026-07-20

**Authors:** Zhefang Wang, Qiu Huang, Jiangang Zhao, Zicheng Lyu, Feng Ju, Bo You, Jie Wang, Qiongzhu Dong, Margarete Odenthal, Alexander Quaas, Kalliope N. Diakopoulos, Hana Algül, Maximilian Reichert, Christiane J. Bruns, Yue Zhao

**Affiliations:** 1 Department of General, Visceral, Thoracic and Transplantation Surgery, University Hospital of Cologne, Cologne, Germany; 2 Department of Plastic and Reconstructive Surgery, Second Affiliated Hospital, School of Medicine, Zhejiang University, Hangzhou, PR China; 3 Institute of Otolaryngology head and neck surgery, Affiliated Hospital of Nantong University, Nantong, PR China; 4 Institute of Pathology, University of Cologne, Faculty of Medicine and University Hospital of Cologne, Cologne, Germany; 5 Key Laboratory of Whole-period Monitoring and Precise Intervention of Digestive Cancer, Shanghai Municipal Health Commission, Minhang Hospital, Fudan University, Shanghai, PR China; 6 Comprehensive Cancer Center München, Institute for Tumor Metabolism, Klinikum rechts der Isar, Technical University of Munich, School of Medicine and Health, Munich, Bavaria Germany; 7 Department of Medicine II, Klinikum Rechts der Isar, Technical University of Munich, Munich, Germany

**Keywords:** Cancer metabolism, Pancreatic cancer

## Abstract

Pancreatic ductal adenocarcinoma (PDAC) remains a highly lethal malignancy due to its aggressive biology and therapeutic resistance. Lysine-specific demethylase 1 (LSD1), an epigenetic regulator, is overexpressed in PDAC and linked to poor prognosis, yet its context-dependent roles in metabolic subtypes and chemoresistance remain undefined. Here, we show that LSD1 knockdown has opposing, subtype-specific effects on chemotherapeutic responses: it sensitized RSK-subtype cells (L3.6pl, PANC-1) to chemotherapy but induced resistance in KRAS-subtype cells (BxPC-3, TBO368). Integrated analyses revealed mitochondrial dysfunction and defective mitophagy as hallmarks distinguishing KRAS- from RSK-subtype PDAC. Critically, mitochondrial targeting through respiratory modulation or mitophagy manipulation overrides LSD1-mediated subtype-specific chemoresistance, establishing mitochondrial fitness as the mechanistic determinant. Mechanistically, LSD1 transcriptionally regulates GLS2 to drive glutamine metabolic reprogramming, promoting reductive carboxylation in KRAS-subtype cells and oxidative metabolism in RSK-subtype cells. Our work establishes the LSD1-GLS2 axis as a metabolic switch controlling PDAC chemosensitivity and provides a framework for subtype-specific therapeutic strategies.

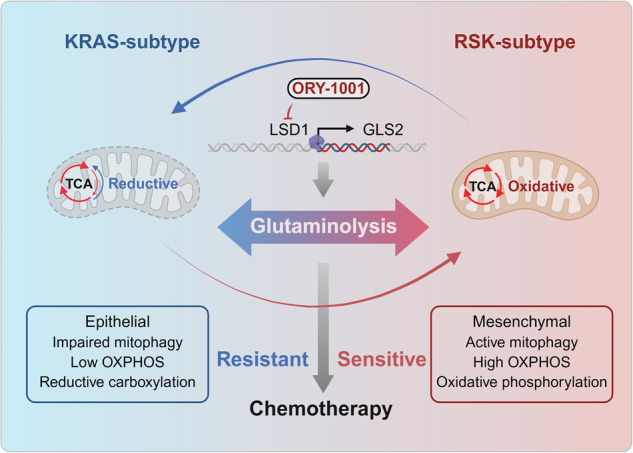

## Introduction

Pancreatic ductal adenocarcinoma (PDAC) is one of the most lethal malignancies in the world and ranks as the sixth leading cause of cancer death, accounting for 5% of all cancer deaths worldwide [1, 2]. The 5-year survival rate of PDAC is less than 10%, while the mortality rate continues to rise gradually [1]. To date, chemotherapy remains the primary approach to managing PDAC, aside from curative surgery. However, acquired resistance to chemotherapy remains the most significant challenge in the management of PDAC [3, 4]. While the underlying mechanisms remain elusive, they are closely linked to intertumor and intratumor heterogeneity in PDAC [5, 6]. Recent studies have classified PDAC into two main molecular subtypes: the basal-like/squamous subtype, associated with poor prognosis and chemoresistance, and the classical subtype, which demonstrates relatively better outcomes [3]. Concurrently, metabolic heterogeneity and reprogramming have emerged as critical determinants of chemotherapy resistance in PDAC. Metabolomic profiling studies have classified PDAC tumors into several distinct metabolic subtypes, including glycolytic, lipogenic, and quiescent [7–10]. The glycolytic subtype is associated with the basal-like subtype and chemoresistance, whereas the lipogenic subtype aligns with the classical subtype [11, 12]. However, classifications based on mitochondrial status (functional or dysfunctional) remain unexplored in PDAC. Functionally intact mitochondria exhibit active oxidative phosphorylation (OXPHOS) and canonical TCA cycling, whereas dysfunctional mitochondria display impaired OXPHOS with preferential flux through reductive carboxylation [13, 14]. These distinct metabolic programs may contribute to the differential therapeutic responses observed across cancers. For instance, tumors with high OXPHOS demonstrate increased chemotherapy sensitivity in ovarian cancer [15, 16], glioblastoma [17], and triple-negative breast cancer (TNBC) [18]. It would be valuable to investigate whether mitochondrial status defines PDAC phenotypes and contributes to subtype-specific behaviors, particularly in the context of chemotherapeutic response.

Lysine-specific demethylase 1 (LSD1), an epigenetic regulator, regulates gene expression by demethylating histone H3 at lysine 4 (H3K4me1/2) or lysine 9 (H3K9me1/2), thereby modulating transcriptional activation or repression [19]. LSD1 has emerged as a key oncogenic driver in multiple cancers, promoting proliferation, metastasis, immune evasion, and chemoresistance [20–25]. These functions are potentially mediated through LSD1’s critical regulation of metabolic reprogramming at the epigenetic-metabolic interface [26]. Notably, prior investigations of LSD1 in cancer metabolism have focused predominantly on glycolysis [27, 28] and lipid metabolism [29–31], while its regulatory role in glutamine metabolism remains largely unexplored. Glutamine is a critical nutrient that drives diverse metabolic and biosynthetic processes supporting tumor growth [32]. In glutamine-dependent cancer cells, over 50% of tricarboxylic acid (TCA) metabolites derive from glutamine through glutaminolysis. This anaplerotic flux is primarily regulated by glutaminases encoded by two distinct genes: glutaminase (GLS) and glutaminase 2 (GLS2) [33]. Due to PDAC’s glutamine addiction, targeting glutamine metabolism has emerged as a promising strategy to overcome chemoresistance [34]. In PDAC, LSD1 was reported to be upregulated in tumor and linked to tumor growth and poor prognosis [35, 36]. However, the role of LSD1 in glutamine metabolic reprogramming and chemoresistance, particularly across distinct PDAC subtypes, remains unexplored.

In this study, we investigate the role of LSD1 in PDAC chemoresistance, with a focus on its interaction with metabolic heterogeneity. We demonstrate that LSD1 exhibits subtype-specific effects on chemotherapeutic response: its ablation sensitizes RSK-subtype cells (characterized by mesenchymal features and OXPHOS) to chemotherapy but induces resistance in KRAS-subtype cells (marked by epithelial features and mitochondrial dysfunction). This context-dependent role represents a crucial consideration for precision therapies in PDAC.

## Results

### LSD1 is a prognostic marker in PDAC

**Fig. 1:**
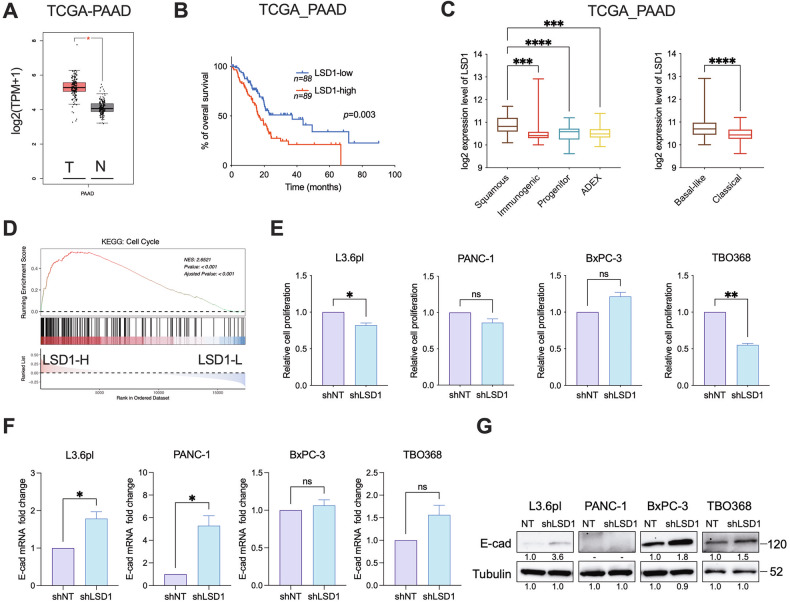
LSD1 is a prognostic marker in PDAC, regulating proliferation and E-cadherin repression. **A** LSD1 expression analysis in PDAC using GEPIA tool. LSD1 expression was elevated in PDAC tissues as compared to normal tissues. **B**–**D** TCGA-PAAD dataset analysis. **B** Kaplan-Meier survival analysis shows high LSD1 expression is associated with poor survival of PDAC patients. Log-rank. **C** Differential LSD1 expression across PDAC molecular subtypes, showing significant enrichment in squamous/basal-like tumors. Data are presented as a box-and-whisker plot (Min to Max). Group comparisons were performed using unpaired two-tailed Student’s *t* tests. For analysis involving three or more groups, one-way ANOVA with Sidak’s correction was applied. **D** Gene set enrichment analysis (GSEA) revealed significant enrichment of KEGG cell cycle pathway genes in the LSD1-high group. **E** Cell proliferation assay showing growth inhibition following LSD1 knockdown in all cell lines except BxPC-3. **F**, **G** Consistent upregulation of E-cadherin at both transcriptional (qRT-PCR) and translational (western blot) levels following LSD1 knockdown across all PDAC cell lines. Tubulin served as a loading control. The experiments were carried out in biological triplicates. Statistical significance was determined by a paired two-tailed Student’s *t* test, otherwise indicated. *p < 0.05, **p < 0.01, ***p < 0.001, ****p < 0.0001, ns: non-significant, *p* > 0.05.

To assess the expression patterns and functional characteristics of LSD1 in PDAC, we analyzed transcriptomic data from the Cancer Genome Atlas (TCGA-PAAD) cohort and the Clinical Proteomic Tumor Analysis Consortium (CPTAC-PDA) database, and single-cell RNA-sequencing data from GSE212966 and GSE202051. LSD1 expression was elevated in PDAC tumors as compared to normal tissues (Fig. 1A and Figure S1A). Survival analysis revealed that higher expression of LSD1 was associated with poor overall survival of PDAC in both TCGA and CPTAC data (Fig. 1B and Figure S1B). To further evaluate the prognostic value of LSD1 in tumor, we assessed the expression of LSD1 in different PDAC molecular subtypes. Results revealed that the expression of LSD1 was significantly higher in the basal-like/squamous subtype compared to others (Fig. 1C and Figure S1C). Critically, LSD1 showed higher expression in patients with poor response to neoadjuvant therapy as compared to those with minimal-moderate response (Figure S1D). To further investigate the function of LSD1 in PDAC, stable LSD1 knockdown cells were established in four PDAC cell lines. The knockdown efficiency was confirmed by western blot (Figure S1E). To validate the successful establishment of LSD1 knockdown functionally, we initially assessed its well-documented roles in various cancers, notably its promotion of cellular proliferation [21, 36] and repression of E-cadherin expression [37, 38]. Gene set enrichment analysis (GSEA) revealed significant enrichment of the cell cycle pathway in LSD1-high group within the TCGA dataset (Fig. 1D). We observed proliferation inhibition after knockdown of LSD1 in L3.6pl and TBO368, but no difference was observed in PANC-1 and BxPC-3 (Fig. 1E). Additionally, consistent elevation of E-cadherin expression occurred across all four cell lines following LSD1 knockdown (Fig. 1F, 1G). However, the fold-change increase at the transcript level was less pronounced in BxPC-3 and TBO368 relative to L3.6pl and PANC-1 (Fig. 1F). Notably, L3.6pl and PANC-1 cells exhibited significantly higher Vimentin expression, while BxPC-3 and TBO368 cells showed elevated E-cadherin levels (Figure S1F), suggesting distinct mesenchymal and epithelial phenotypes, respectively. These likely explain the attenuated E-cadherin response to LSD1 knockdown in cells with epithelial phenotypes. In summary, these findings establish LSD1 as a prognostic biomarker in PDAC and corroborate its reported function of promoting cellular proliferation while repressing E-cadherin expression in other cancer contexts.

### LSD1 exhibits context-dependent roles in PDAC chemotherapeutic response

**Fig. 2:**
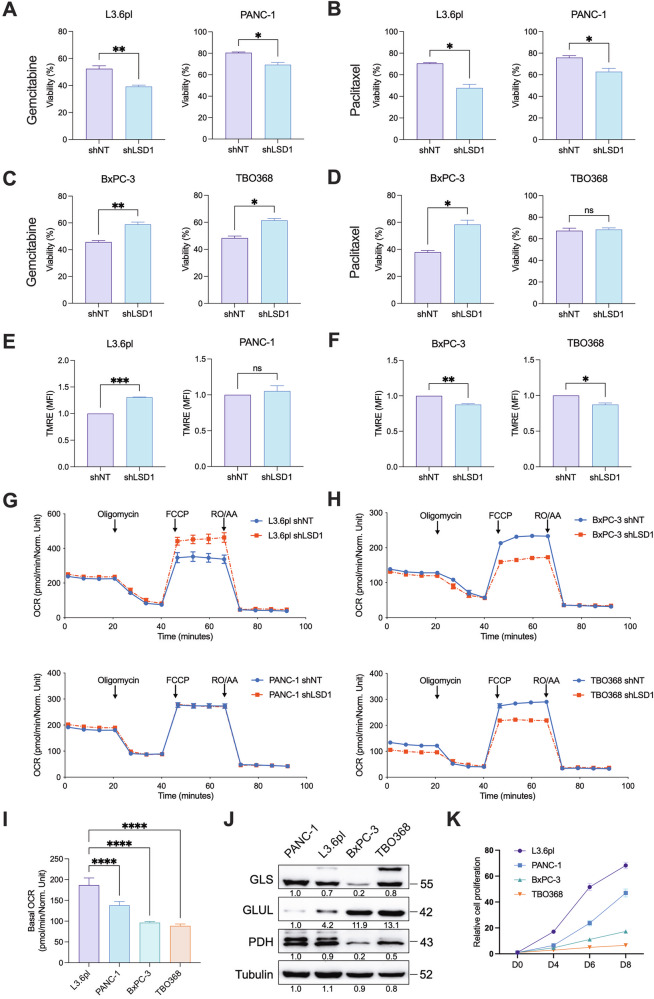
LSD1 exhibits context-dependent roles in PDAC chemotherapeutic response. **A**–**D** Chemosensitivity analysis following LSD1 knockdown. Cells were treated with either gemcitabine (3 ng/ml for L3.6pl and 400 ng/ml for others) or paclitaxel (25 nM) for 48–72 h, then assessed by Annexin V/DAPI apoptosis assay. LSD1 knockdown significantly sensitized L3.6pl and PANC-1 to both chemotherapeutics while inducing resistance in BxPC-3 and TBO368. Data represent mean ± SEM of three independent experiments. **E**, **F** Mitochondrial membrane potential (ΔΨm) analysis. TMRE was used to determine the mitochondrial membrane potential (ΔΨm). Mean fluorescence intensity (MFI) quantification revealed increased ΔΨm in L3.6pl and PANC-1 but decreased potential in BxPC-3 and TBO368. **G**, **H** Mitochondrial respiration profiling using Seahorse XF Mito Stress Test. Six replicates for each group. LSD1 knockdown enhanced mitochondrial respiration in L3.6pl, showed no change in PANC-1, but reduced in BxPC-3 and TBO368. **I** Basal oxygen consumption rate (OCR) of four PDAC cells was demonstrated as a bar chart. One-way ANOVA. **J** Western blot analysis of GLS, GLUL, and PDH. Tubulin served as a loading control. **K** Cell proliferation assay revealed that L3.6pl and PANC-1 cells proliferated more rapidly than BxPC-3 and TBO368 cells. The experiments were carried out in biological triplicate. Statistical significance was determined by a paired two-tailed Student’s *t* test. *p < 0.05, **p < 0.01, ***p < 0.001, ns: non-significant, *p* > 0.05.

As LSD1 is enriched in the chemotherapy-resistant basal-like/squamous PDAC subtype, we speculate that LSD1 may promote chemoresistance. Intriguingly, LSD1 knockdown yielded divergent responses to chemotherapy across the four PDAC cell lines. Specifically, LSD1 knockdown sensitized L3.6pl and PANC-1 cells to gemcitabine and paclitaxel (Fig. 2A, 2B) but increased resistance in BxPC-3 cells to both drugs and in TBO368 cells to gemcitabine (Fig. 2C, 2D). To further validate this effect, we generated stable LSD1 overexpression cell lines (Figure S2A). Consistent with the knockdown phenotype, exogenous LSD1 expression recapitulated divergent chemotherapeutic responses: while inducing modest chemoresistance in L3.6pl and PANC-1 (no statistical significance achieved), it significantly enhanced chemosensitivity in BxPC-3 and TBO368 (Figure S2B, S2C). This divergent regulation further validates LSD1’s context-dependent roles in PDAC chemotherapeutic response. Since mitochondria are closely associated with chemotherapy-induced apoptosis and play pivotal roles in chemoresistance [39, 40], we further profiled mitochondrial functions in these cells. Accordingly, mitochondrial function exhibited heterogeneity in these four cell lines. In L3.6pl and PANC-1 cells, knockdown of LSD1 resulted in either elevated or unchanged mitochondrial membrane potential (ΔΨm) and mitochondrial respiration (Fig. 2E, 2G). By contrast, in BxPC-3 and TBO368 cells, LSD1 knockdown led to decreased mitochondrial membrane potential (ΔΨm) and mitochondrial respiration (Fig. 2F, 2H). Thus, we hypothesize that these cells may represent different metabolic subtypes of PDAC. A significantly higher basal oxygen consumption rate (OCR) was observed in L3.6pl and PANC-1 cells relative to BxPC-3 and TBO368 cells (Fig. 2I), indicating strengthened mitochondrial respiration activity. Supportively, L3.6pl and PANC-1 cells displayed increased expression of GLS and pyruvate dehydrogenase (PDH) but reduced glutamine synthetase (GLUL) levels compared to BxPC-3 and TBO368 cells (Fig. 2J), suggesting enhanced mitochondrial tricarboxylic acid (TCA) cycle influx. Furthermore, proliferation assays revealed that L3.6pl and PANC-1 cells proliferated more rapidly than BxPC-3 and TBO368 cells (Fig. 2K). Taken together, these data suggest LSD1 exhibits subtype-specific, context-dependent roles in PDAC chemotherapeutic response, which may correspond to the underlying metabolic subtypes of the disease.

### KRAS-RSK subtyping determines the context-dependent roles of LSD1 in PDAC

**Fig. 3:**
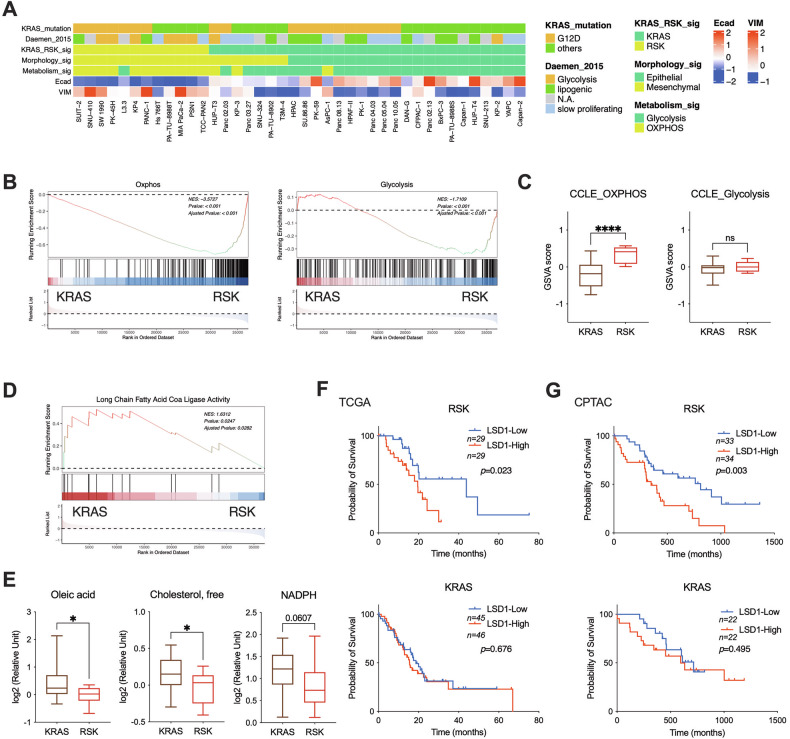
KRAS-RSK subtyping determines the context-dependent roles of LSD1 in PDAC. **A**–**C** CCLE data analysis. **A** Subtype stratification of PDAC cell lines. Unsupervised clustering of 40 CCLE PDAC lines aligned to established molecular subtypes. KRAS mutation status and EMT markers (E-cadherin and Vimentin) are annotated. **B** GSEA demonstrated significant enrichment of OXPHOS and Glycolysis signatures in the RSK subtype. **C** GSVA demonstrated significantly elevated OXPHOS score in RSK subtype; no difference was observed for glycolysis (*n* = 28 in KRAS, *n* = 12 in RSK). **D** GSEA demonstrated significant enrichment of long-chain fatty acid CoA ligase activity signature in the KRAS subtype. **E** Metabolomics data from Daemen et al. showed higher levels of oleic acid, cholesterol, and NADPH in KRAS-subtype cells (*n* = 22 in KRAS, *n* = 10 in RSK). **F**–**G** Kaplan-Meier survival analysis by KRAS-RSK subtypes. Patients with PDAC were stratified using unsupervised hierarchical clustering based on the KRAS_RSK_sig in (**F**) TCGA-PAAD and (**G**) CPTAC-PDA cohorts. Log-rank. Statistical significance was determined by unpaired two-tailed Student’s *t* test otherwise indicated. *p < 0.05, ****p < 0.0001, ns: non-significant, *p* > 0.05.

To understand the differential chemotherapeutic responses to LSD1 knockdown, we sought to classify the four PDAC cell lines according to established molecular subtypes. Notably, the subtyping framework proposed by Yuan et al. [41] closely paralleled our observations: their classification associating KRAS-dependent lines with epithelial/glycolytic phenotypes and RSK-dependent lines with mesenchymal/OXPHOS phenotypes aligned with the distinct morphological and metabolic profiles of our four PDAC cell lines. Consequently, we propose that L3.6pl and PANC-1 represent the RSK/mesenchymal/OXPHOS subtype, while BxPC-3 and TBO368 correspond to the KRAS/epithelial/glycolytic subtype. To extend this classification to a broader cohort, we analyzed RNA-seq data from 40 PDAC cell lines from the CCLE database. Unsupervised hierarchical clustering using Yuan et al.’s signatures identified distinct subtypes. Consistent with previous findings, L3.3 (the parental cell line of L3.6pl) and PANC-1 clustered with the RSK/mesenchymal/OXPHOS subtype, while BxPC-3 aligned with the KRAS/epithelial/glycolytic subtype (Fig. 3A). These subtypes were further distinguishable by their differential expression of E-cadherin and Vimentin (Fig. 3A). Both GSEA and gene set variation analysis (GSVA) results demonstrated OXPHOS pathway enrichment in the RSK subtype (Fig. 3B, 3C). Surprisingly, GSEA analysis revealed enrichment of the glycolysis pathway in the RSK subtype but not in the KRAS subtype (Fig. 3B), whereas GSVA scores showed no significant difference between the two groups (Fig. 3C). This discrepancy likely reflects the distinct analytical approaches of each method: GSEA detects coordinated expression among leading-edge genes, while GSVA evaluates overall pathway activity. In addition, basal extracellular acidification rate (ECAR) was also higher in L3.6pl and PANC-1 compared to BxPC-3 and TBO368 (Figure S2D). Supporting these observations, metabolomic subtyping by Daemen et al. [8] classified most RSK-subtype cell lines as “glycolytic”, whereas KRAS-subtype lines predominantly fell into “lipogenic” or ‘slow-proliferating’ categories (Fig. 3A). Together, these findings demonstrate that the RSK subtype exhibits heightened metabolic plasticity, characterized by concurrent activation of both OXPHOS and glycolytic pathways. In contrast, the KRAS subtype displays a distinct metabolic profile marked by enriched long-chain fatty acid CoA ligase activity (Fig. 3D) and increased accumulation of oleic acid, cholesterol, and NADPH (Fig. 3E), suggesting an alternative mitochondrial metabolism such as reductive carboxylation-fueled lipogenesis [42].

To determine whether the KRAS-RSK subtyping best recaptures the divergent roles of LSD1 in PDAC, we performed Kaplan-Meier survival analyses using TCGA-PAAD and CPTAC-PDA datasets, stratifying patients by KRAS_RSK_sig, Morphology_sig, and Metabolism_sig classifications (Figure S3A, S3C). Strikingly, in both datasets, LSD1 expression correlated with poor prognosis specifically in the RSK subtype when using KRAS_RSK_sig classification, while showing no significant association in the KRAS subtype (Fig. 3F, 3G). In contrast, neither Morphology_sig nor Metabolism_sig subtyping effectively distinguished these differential prognostic roles of LSD1 (Figure S3B, S3D). Noteworthy, the RSK subtype showed a higher prevalence of KRAS-G12D mutations (7/12 vs 12/28 cell lines) compared to the KRAS subtype (Fig. 3A), suggesting that KRAS mutants may take part in subtype determination. Interestingly, while L3.6pl and PANC-1 harbor KRAS G12D mutations, TBO368 carries KRAS G12R and BxPC-3 is KRAS wild-type. However, additional survival analyses stratified by KRAS mutation status did not reach statistical significance in any subgroup (Figure S3B, S3D). Together, these data indicate that KRAS-RSK subtypes dictate the context-dependent roles of LSD1 in PDAC.

Interestingly, although LSD1 is enriched in basal-like/squamous PDAC, its expression levels were comparable between KRAS and RSK subtypes in both cell lines and tumor tissues (Figure S3E). Similarly, we observed no differences in expression of GATA6 and KRT17 (key markers of classical and basal-like subtypes, respectively) between KRAS and RSK subtypes (Figure S3F), underscoring their distinct molecular taxonomy. Furthermore, we analyzed single-cell RNA sequencing (scRNA-seq) data from PDAC [43]. Trajectory analysis demonstrated that KRAS and RSK-subtype markers localized to opposite ends of the pseudotime continuum, supporting their distinct and conserved molecular features in PDAC pathogenesis (Figure S3G, S3H).

To further validate our findings, we selected representative cell lines from each subtype (MiaPaCa-2 for the RSK subtype and HPAF-II for the KRAS subtype) for additional LSD1 knockdown experiments (Figure S3I). Replicating our initial observations, RSK-subtype MiaPaCa-2 cells demonstrated enhanced chemosensitivity upon LSD1 knockdown, while KRAS-subtype HPAF-II cells showed no significant response (Figure S3J). These results collectively indicate that the subtype-specific chemotherapeutic responses following LSD1 knockdown are fundamentally linked to the intrinsic mitochondrial metabolic differences between KRAS and RSK subtypes in PDAC.

### Mitochondrial dysfunction and defective mitophagy as hallmarks distinguish KRAS-RSK subtypes of PDAC

**Fig. 4:**
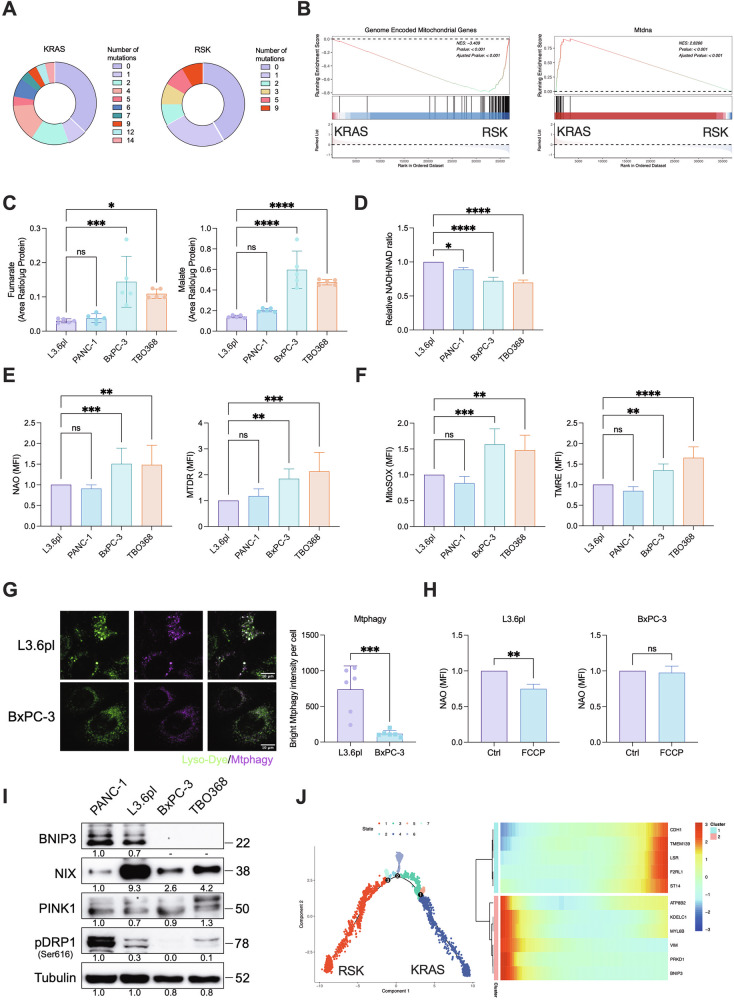
Mitochondrial dysfunction and mitophagy deficiency serve as hallmarks that distinguish KRAS-RSK subtypes of PDAC. **A**, **B** CCLE data analysis. **A** mtDNA mutational landscape. Pie charts quantify mtDNA mutation distribution in KRAS- vs. RSK-subtypes. **B** GSEA of mitochondrial gene signatures. Nuclear-encoded mitochondrial genes are enriched in the RSK-subtype, while mtDNA-encoded genes are enriched in the KRAS-subtype. **C** Metabolomics reveals fumarate/malate accumulation in KRAS-subtype lines (BxPC-3, TBO368) vs. RSK-subtype (L3.6pl, PANC-1). Five replicates for each group. One-way ANOVA. **D** NADH/NAD⁺ ratios are reduced in BxPC-3 and TBO368. One-way ANOVA. **E** NAO and MitoTracker Deep Red (MTDR) staining show increased mitochondrial mass in BxPC-3 and TBO368. MFI quantification. One-way ANOVA. **F** TMRE and MitoSOX staining show increased mitochondrial membrane potential and mtROS in BxPC-3 and TBO368. MFI quantification. One-way ANOVA. **G** Baseline mitophagy. Mtphagy/Lyso dye co-staining shows reduced mitophagic flux in BxPC-3 vs. L3.6pl. Unpaired *t*-test. **H** FCCP-induced mitophagy was determined by NAO staining. **I** Western blot analysis of mitophagy-related proteins BNIP3, NIX, PINK1, and p-DRP1 (Ser616). Tubulin served as a loading control. **J** Trajectory analysis of Peng’s scRNA-seq data. Pseudotime heatmap shows BNIP3 expression positively correlates with RSK-subtype differentiation markers. The experiments were carried out in biological triplicate. Statistical significance was determined by paired two-tailed Student’s *t* test, otherwise indicated. *p < 0.05, **p < 0.01, ***p < 0.001, ****p < 0.0001, ns: non-significant, *p* > 0.05.

To elucidate the metabolic distinctions between KRAS and RSK subtypes, we performed comprehensive molecular analyses. Mutation profiling of CCLE data revealed significant differences in mitochondrial DNA (mtDNA) mutational burden between subtypes. While >50% of KRAS-subtype cell lines carried multiple mtDNA mutations, most RSK-subtype lines showed ≤1 mtDNA mutation (Fig. 4A). Notably, mtDNA mutations predominantly affected Complex I genes, with BxPC-3 exhibiting characteristic missense mutations in MT-ND4 and MT-ND5. Additionally, GSEA and GSVA demonstrated preferential enrichment of nuclear-encoded mitochondrial genes in the RSK subtype versus mtDNA-encoded genes in the KRAS subtype (Fig. 4B and Figure S4A), suggesting distinct mitonuclear genetic regulation [44]. Pseudotime trajectory analysis of single-cell data (Peng et al.) confirmed this observation, with mtDNA-encoded and nuclear-encoded mitochondrial genes clustering at opposite ends of the developmental continuum, corresponding to KRAS and RSK subtypes respectively (Figures S3H, S4C). Together, these results suggest that KRAS-subtype cells may be characterized by dysfunctional mitochondria. Additionally, metabolomic profiling of TCA cycle intermediates demonstrated significant accumulation of fumarate and malate in KRAS-subtype cell lines (BxPC-3 and TBO368; Fig. 4C). Meanwhile, NADH/NAD⁺ ratio was significantly decreased in KRAS-subtype cell lines (Fig. 4D), corroborated by independent metabolomics data from Daemen et al. (Figure S4B). These results indicate that KRAS-subtype cells (BxPC-3 and TBO368) exhibit mitochondrial dysfunctional characterized by mtDNA instability, mitonuclear imbalance, accumulated TCA intermediates, and reduced NADH/NAD+ ratio.

Intriguingly, despite exhibiting elevated basal OCR, L3.6pl and PANC-1 displayed reduced mitochondrial mass compared to BxPC-3 and TBO368, as quantified by both Nonyl Acridine Orange (NAO) and MitoTracker Deep Red (MTDR) staining (Fig. 4E). Furthermore, these RSK-subtype cells demonstrated reduced mitochondrial membrane potential (ΔΨm) and mitochondrial reactive oxygen species (ROS) levels (Fig. 4F). Further examination with transmission electron microscopy revealed mitochondria with crowed and disorganized ultrastructure in BxPC-3 and TBO368 (Figure S4D). These results collectively demonstrate that KRAS-subtype cells accumulate dysfunctional mitochondria, suggesting impaired mitophagic clearance as a potential mechanism underlying their metabolic phenotype. To assess mitophagic activity, we performed immunofluorescence staining in L3.6pl and BxPC-3 using a mitophagy-specific dye (Mtphagy dye). BxPC-3 demonstrated markedly reduced basal mitophagy compared to L3.6pl (Fig. 4G). Furthermore, FCCP did not induce significant mitochondrial reduction in BxPC-3 compared to L3.6pl (Fig. 4I). Consistent with these findings, GSEA and GSVA analyses revealed preferential enrichment of mitophagy-related pathways in the RSK subtype (Figure S4A). Western blotting of mitophagy regulators demonstrated markedly lower protein levels of BCL2 interacting protein 3 (BNIP3) and phosphorylated Dynamin-1-like protein (DRP1) in KRAS-subtype cells (Fig. 4I). Given the pronounced subtype-specific divergence in BNIP3 expression, we further examined BNIP3 expression in Peng’s single-cell data. Pseudotime trajectory analysis demonstrated a positive correlation between BNIP3 expression and RSK-subtype differentiation, coupled with an inverse association with KRAS-subtype markers (Fig. 4J), underscoring the critical role that BNIP3 may play in RSK-subtype cells. Additionally, survival analysis revealed that LSD1 expression correlated with poor prognosis specifically in BNIP3-high subgroup, whereas no significant prognostic difference was observed in BNIP3-low subgroup (Figure S3B, S3D). Collectively, these findings suggest that KRAS-subtype PDAC cells exhibit mitochondrial dysfunction and defective mitophagy, which may constitute fundamental determinants of their metabolically distinct phenotypes.

### Mitochondrial targeting overrides LSD1’s subtype-specific regulation of chemotherapeutic response in PDAC subtypes

**Fig. 5:**
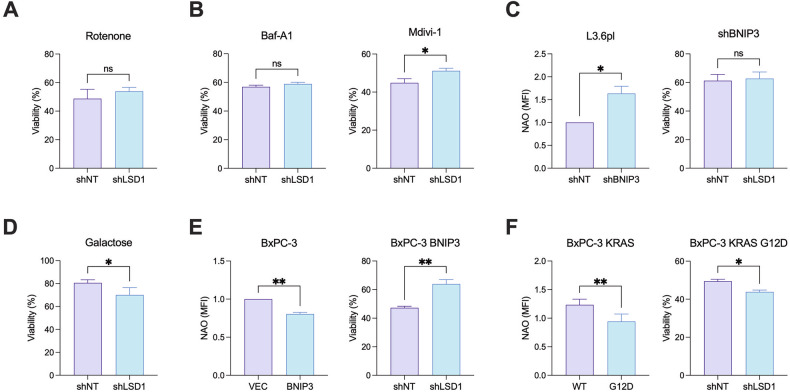
Mitochondrial targeting overrides LSD1’s context-dependent regulation of chemotherapeutic response in PDAC subtypes. **A** Rotenone (100 nM), (**B**) Baf-A1 (1 µM), and Mdivi-1 (25 µM) co-treatment abolished LSD1 knockdown-induced chemosensitivity in L3.6pl. **C** BNIP3 knockdown induced mitochondrial accumulation in L3.6pl and abolished LSD1 knockdown-induced chemosensitivity. **D** Galactose enforced OXPHOS reversed LSD1 knockdown-induced chemoresistance in BxPC-3. **E** BNIP3 overexpression reduced mitochondrial content in BxPC-3 but failed to reverse chemoresistance. **F** KRAS-G12D mutation induced mitochondrial clearance in BxPC-3 and reversed LSD1 knockdown-induced chemoresistance. The experiments were carried out in biological triplicate. Statistical significance was determined by a paired two-tailed Student’s *t* test, otherwise indicated. *p < 0.05, **p < 0.01, ns: non-significant, *p* > 0.05.

Given the distinct mitochondrial phenotypes of KRAS- and RSK-subtypes, we assessed whether targeting mitochondrial function via either electron transport chain (ETC) impairment or mitophagy inhibition, overrides LSD1’s subtype-specific regulation of chemotherapeutic response in RSK-subtype cells. First, Complex I inhibitor rotenone was employed to impair mitochondrial function in RSK-subtype cells. Not surprisingly, rotenone abolished LSD1 knockdown-induced chemosensitivity in L3.6pl (Fig. 5A). Next, mitophagy inhibitors Baf-A1 and Mdivi-1 were applied. Consistently, Baf-A1 abolished LSD1 knockdown-induced chemosensitivity in L3.6pl, while Mdivi-1 even reversed it (Fig. 5B). Given the significance of mitophagy receptor BNIP3 in RSK-subtype cells, we inhibited mitophagy through BNIP3 depletion as well (Figure S5A). BNIP3 knockdown induced mitochondrial accumulation and abolished LSD1 knockdown-induced chemosensitivity in L3.6pl (Fig. 5C). Furthermore, we assessed whether enforced mitochondrial respiration or restoration of mitophagy could do the same in KRAS-subtype cells. Forced OXPHOS dependency via galactose medium was applied and reversed LSD1 knockdown-induced chemoresistance was observed in BxPC-3 (Fig. 5D). In contrast, forced BNIP3 expression (Figure S5A) reduced mitochondrial content in BxPC-3 but failed to reverse LSD1 knockdown-induced chemoresistance (Fig. 5E). Mitochondrial respiration analysis revealed unchanged basal OCR and maximal OCR after BNIP3 expression (Figure S5B). These results validate that mitochondrial functional status determines the mechanisms underlying LSD1’s subtype-specific roles in PDAC chemosensitivity, and mitochondrial targeting may override its regulation.

As BxPC-3 is KRAS wild-type, we also introduced the KRAS-G12D mutation to assess its role in determining the KRAS-RSK phenotype (Figure S5C, D). Compared to wild-type controls, KRAS-G12D significantly altered cell morphology (Figure S5C) and triggered a mesenchymal transition evidenced by Vimentin upregulation and E-cadherin downregulation at both transcriptional and protein level (Figure S5E). Furthermore, KRAS-G12D reduced mitochondrial content in BxPC-3 cells relative to wild-type controls and reversed the LSD1 knockdown-induced chemoresistance observed in parental cells (Fig. 5F and Fig. 2C). These data suggest an important role of KRAS-G12D mutation in KRAS-RSK-subtype determination, particularly in regulating LSD1’s context-dependent functions, though further investigation is required.

### LSD1 shapes chemotherapeutic response in PDAC through GLS2-mediated glutaminolysis reprogramming

**Fig. 6:**
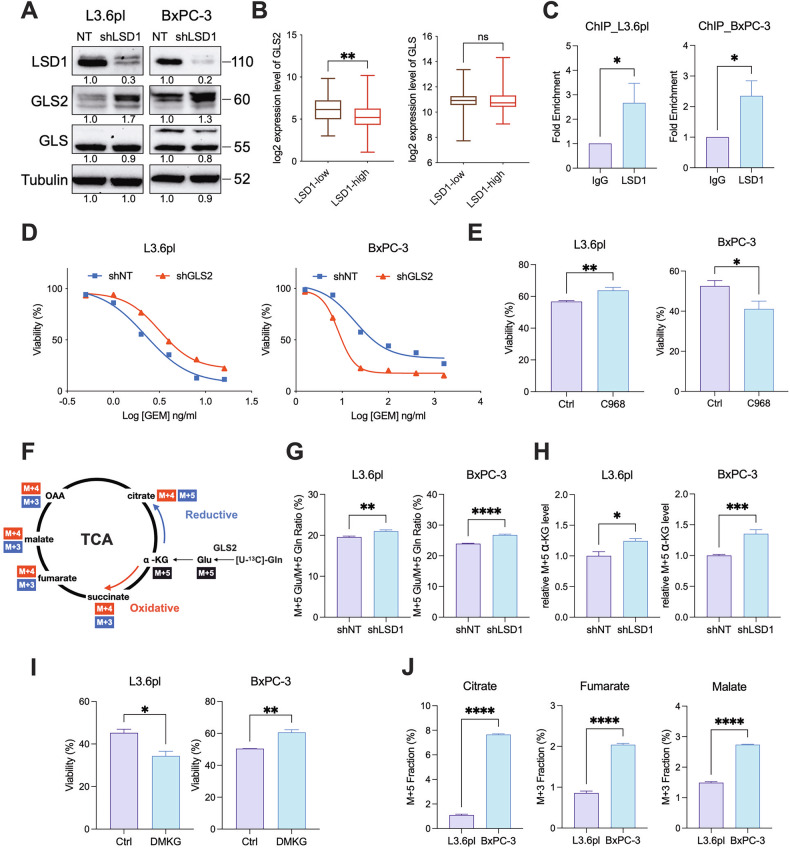
LSD1 shapes chemotherapeutic response in PDAC through GLS2-mediated glutamine metabolic reprogramming. **A** Western blot analysis of LSD1, GLS, and GLS2. Tubulin served as a loading control. **B** TCGA analysis demonstrated significantly elevated GLS2 expression in LSD1-low versus LSD1-high PDAC patients, with no difference in GLS levels. **C** Chromatin immunoprecipitation (ChIP) assays confirmed LSD1 binding at the GLS2 promoter. Paired *t*-test. **D** GLS2 knockdown sensitized BxPC-3 to gemcitabine but increased resistance in L3.6pl. **E** Pharmacological GLS2 inhibition with C968 (5 µM) phenocopied genetic knockdown of GLS2. Paired *t*-test. **F** Schematic model of U-¹³C-Glutamine flux through the TCA cycle. Cells were labeled with 4 mM U-¹³C-Glutaminec for 24 h. Five replicates for each group. **G** M + 5 glutamate/glutamine ratio was increased after LSD1 knockdown. **H** M + 5 α-KG level was elevated after LSD1 knockdown. **I** Supplementation of dimethyl alpha-ketoglutarate (DMKG) sensitizing L3.6pl to gemcitabine while conferring resistance in BxPC-3. Paired *t*-test. **J** Fractions of *M* + 5 citrate, *M* + 3 fumarate, and M + 3 malate were significantly higher in BxPC-3 compared to L3.6pl. The experiments were carried out in biological triplicate. Statistical significance was determined by unpaired two-tailed Student’s *t* test, otherwise indicated. *p < 0.05, **p < 0.01, ***p < 0.001, ****p < 0.0001, ns: non-significant, *p* > 0.05.

Based on our findings, we hypothesized that LSD1 regulates subtype-specific chemotherapeutic response by controlling metabolic reprogramming. Given the central role of glucose and glutamine as mitochondrial carbon sources, we evaluated LSD1’s influence on glycolysis and glutaminolysis. Although prior studies link LSD1 to glycolytic regulation [27, 28], its knockdown did not alter the expression of key enzymes in late-phase glycolysis (LDHA) and pyruvate oxidation (PDH) (Figure S6A). Instead, we identified liver-type glutaminase (GLS2), but not kidney-type glutaminase (GLS), as consistently upregulated upon LSD1 knockdown in both L3.6pl and BxPC-3 cells (Fig. 6A). Consistent with these findings, TCGA analysis demonstrated elevated GLS2 expression in LSD1-low versus LSD1-high PDAC patients, with no significant difference in GLS levels (Fig. 6B). Clinically, higher GLS2 expression correlated with improved overall survival in PDAC patients, while GLS showed no prognostic association (Figure S6B). To define the regulatory mechanism, chromatin immunoprecipitation (ChIP) assays revealed direct binding of LSD1 to the GLS2 promoter (Fig. 6C). Furthermore, LSD1 knockdown increased H3K4me3 enrichment at this locus (Figure S6C), supporting its role as a transcriptional repressor of GLS2. Functional validation using stable GLS2-knockdown cells (Figure S6D) demonstrated that GLS2 depletion enhanced chemoresistance in RSK-subtype L3.6pl but increased chemosensitivity in KRAS-subtype BxPC-3 (Fig. 6D). These subtype-specific responses were recapitulated using the GLS2 inhibitor C968 (Fig. 6E). To directly assess glutamine metabolic flux, we performed isotopic tracing with U-^13^C-glutamine (Fig. 6F). LSD1 knockdown enhanced glutaminase activity in both subtypes, evidenced by increased glutamate-to-glutamine ratio (Fig. 6G and Figure S6E) and elevated α-ketoglutarate (α-KG) levels (Fig. 6H and Figure S6F). These changes were observed in both the M + 5 isotopologue alone and the total labeled fraction (M + 1 to M + 5), indicating enhanced glutamine entry into the TCA cycle. Exogenous α-KG supplementation phenocopied subtype-specific chemosensitivity, confirming its functional relevance (Fig. 6I). We next asked how enhanced glutaminolysis elicits opposing responses across PDAC subtypes. Given that mitochondrial dysfunction promotes reductive carboxylation [13, 14], we hypothesized that RSK- and KRAS-subtypes exhibit distinct metabolic flux. Supportively, established reductive carboxylation indicators, namely citrate/α-KG ratio and NADPH/NADP+ ratio, were both elevated in BxPC-3 relative to L3.6pl (Figure S6G). More directly, isotope tracing revealed substantially higher fractions of M + 5 citrate, M + 3 fumarate, and M + 3 malate in BxPC-3 (Fig. 6J), confirming preferential reductive flux in KRAS-subtype BxPC-3 cells. Collectively, these data demonstrate that GLS2-mediated glutaminolysis drives preferential utilization of reductive carboxylation in KRAS-subtype BxPC-3 while promoting oxidative metabolism in RSK-subtype L3.6pl. Our findings establish LSD1/GLS2 axis-mediated reprogramming of glutamine metabolism as a critical mechanism underlying subtype-specific regulation of chemotherapeutic response in PDAC.

### Inhibition of LSD1 recapitulates subtype-specific chemosensitivity in organoids

**Fig. 7:**
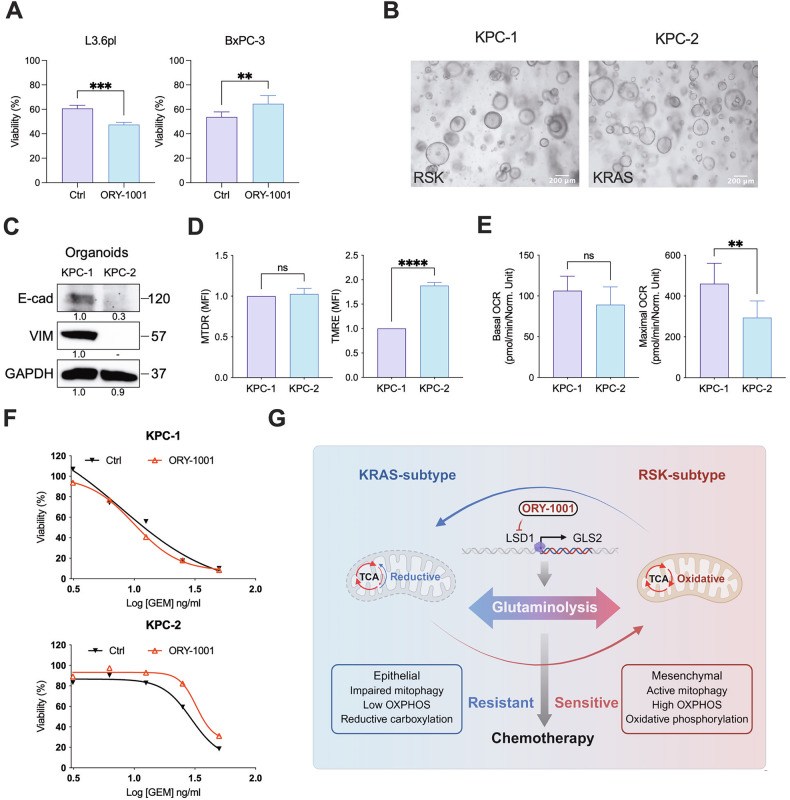
Inhibition of LSD1 recapitulates subtype-specific chemosensitivity in organoids. **A** ORY-1001 (50 µM) showed synergy with gemcitabine in L3.6pl whereas antagonizing gemcitabine in BxPC-3. **B** Brightfield imaging of KPC-derived organoids. **C** Western blot analysis of E-cadherin and Vimentin in KPC-derived organoids. GAPDH served as loading control. **D** MTDR staining shows similar mitochondrial mass in KPC-1 and KPC-2. TMRE staining shows increased mitochondrial membrane potential in KPC-2. MFI quantification. **E** Mitochondrial respiration profiling using Seahorse XF Mito Stress Test. KPC-2 shows reduced mitochondrial respiration compared to KPC-1. **F** ORY-1001 (50 µM) modestly enhanced gemcitabine sensitivity in KPC-1 organoids while significantly inducing resistance in KPC-2 organoids. **G** Schematic diagram illustrating LSD1/GLS2 axis-mediated subtype-specific chemotherapeutic response in PDAC. The figure was created on BioRender.com. The experiments were carried out in biological triplicate. Statistical significance was determined by a paired two-tailed Student’s *t* test, otherwise indicated. **p < 0.01, ***p < 0.001.

Following characterization of LSD1’s subtype-specific role in chemoresistance, we subsequently investigated the functional consequences of pharmacological LSD1 inhibition in human PDAC cell lines and murine organoid models. Consistently, LSD1 Inhibitor ORY-1001 synergized with gemcitabine in RSK-subtype L3.6pl but antagonized its efficacy in KRAS-subtype BxPC-3 (Fig. 7A). To better model in vivo subtype heterogeneity, we established two Pdx1-Cre; Kras^G12D/+^;p53^R172H/+^ (KPC)-derived organoid lines (Fig. 7B). Based on expression of E-cadherin and Vimentin, KPC-1 was classified as RSK-subtype, while KPC-2 was designated KRAS-subtype (Fig. 7C). Consistent with this classification, KPC-2 exhibited increased mitochondrial membrane potential (ΔΨm) (Fig. 7D) but reduced mitochondrial respiration (Fig. 7E), indicating defective mitochondrial function compared to KPC-1. Critically, LSD1 inhibition with ORY-1001 produced subtype-specific responses in KPC-derived organoids as well: it modestly enhanced gemcitabine sensitivity in RSK-subtype KPC-1 organoids while significantly inducing resistance in KRAS-subtype KPC-2 organoids (Fig. 7F). Therefore, LSD1 inhibition shows subtype-specific chemotherapeutic responses in PDAC (Fig. 7G).

## Discussion

Our study establishes LSD1 as a critical epigenetic-metabolic regulator in PDAC whose therapeutic targeting requires subtype stratification. While LSD1 promotes proliferation and suppresses E-cadherin, consistent with its known oncogenic functions [21, 37], we reveal its previously unexplored context-dependent role in chemoresistance. Strikingly, LSD1 ablation sensitizes RSK-subtype cells to chemotherapy while inducing resistance in KRAS-subtype cells.

This divergence aligns with the KRAS/RSK subtyping framework initially proposed by Singh et al. [45] and subsequently refined in KRAS-mutant cancers (including lung cancer, colon cancer, and PDAC) [41]. Although classical/basal-like subtyping remains a cornerstone of PDAC classification, our findings suggest that KRAS/RSK subtyping more accurately captures the divergent roles of LSD1, as demonstrated by survival analysis. While KRAS/classical subtypes share epithelial features (e.g., E-cadherin expression) and RSK/basal-like subtypes exhibit mesenchymal traits (e.g., Vimentin expression), key lineage markers of classical/basal-like subtypes (GATA6, KRT17) [46, 47] show no differential expression between KRAS and RSK subtypes, underscoring their distinct molecular taxonomy. This complexity underscores PDAC’s molecular heterogeneity across existing subtyping frameworks and emphasizes the necessity of defining molecular context prior to therapeutic decision-making.

The KRAS and RSK subtypes exhibit distinct metabolic profiles. RSK-subtype cells maintain functional mitochondria with active OXPHOS and a hybrid metabolic state utilizing both oxidative metabolism and glycolysis. In contrast, KRAS-subtype cells display mitochondrial dysfunction marked by mtDNA mutations, accumulation of TCA intermediates, NADH/NAD+ imbalance, and defective mitophagy. This dysfunction forces reliance on alternative pathways, including reductive carboxylation [13, 14]. Given these distinct mitochondrial profiles, we interrogated subtype plasticity by targeting mitochondrial respiration. In RSK-subtype L3.6pl cells, Complex I inhibition (impairing OXPHOS) abolished LSD1 knockdown-induced chemosensitivity. Conversely, in KRAS-subtype BxPC-3 cells, galactose substitution (enforcing OXPHOS dependency) reversed chemoresistance associated with LSD1 knockdown. These findings establish mitochondrial dysfunction as a defining feature of the KRAS subtype and demonstrate that metabolic modulation could potentially override subtype-specific therapeutic responses.

Mitochondrial dysfunction and impaired mitophagy are intimately linked processes. Compromised mitophagy exacerbates mitochondrial dysfunction by permitting damaged organelle accumulation [48]. In this study, we identified BNIP3 as a key mediator of mitophagy in RSK-subtype PDAC. BNIP3 expression is upregulated during early premalignant stages of PDAC but is frequently downregulated during tumor progression. This downregulation has been functionally linked to intrinsic gemcitabine resistance in PDAC cell lines [49, 50]. Mechanistically, BNIP3 deficiency impairs mitochondrial clearance, resulting in persistent damaged mitochondria, elevated mitochondrial ROS, redox imbalance, and pro-survival signaling that collectively promote chemoresistance [51, 52]. While BNIP3 depletion abrogated LSD1 knockdown-induced chemosensitivity in RSK-subtype L3.6pl cells, BNIP3 restoration in KRAS-subtype BxPC-3 cells failed to reverse LSD1 knockdown-induced chemoresistance. These results indicate that BNIP3-mediated mitophagy is necessary but insufficient to rescue mitochondrial function in KRAS-subtype PDAC, possibly due to irreparable Complex I defects from inherited mtDNA mutations.

Since KRAS mutations are primary drivers of PDAC tumorigenesis and closely related to mitochondrial dynamics [53, 54], we introduced the KRAS-G12D mutation into BxPC-3 cells. The introduction of KRAS-G12D reversed the chemoresistance phenotype induced by LSD1 ablation in parental BxPC-3 cells. Nevertheless, KRAS-G12D mutation alone may be insufficient to dictate subtype identity, as over 40% of KRAS-subtype cells identified here harbored KRAS-G12D mutations, such as HPAF-II. This discrepancy likely reflects complexities in KRAS copy number variation and allelic imbalance (mutant-to-wild-type allele ratios), underscoring the multifactorial genomic regulation of PDAC subtype identity [6, 55].

While prior studies focused on LSD1’s regulation of glycolysis and lipid metabolism, we establish its critical role in glutaminolysis through GLS2 repression. In this study, we demonstrate that LSD1 governs glutamine metabolism by transcriptionally regulating GLS2 expression. Both genetic and pharmacologic inhibition of GLS2 recapitulated the subtype-specific chemosensitivity patterns observed with LSD1 ablation. Although LSD1 ablation enhances glutaminolysis in both subtypes, mitochondrial status determines the metabolic fate of glutamine flux. Isotopic metabolic flux analysis suggests preferential glutamine flux through oxidative metabolism in RSK-subtype versus through reductive carboxylation in KRAS-subtype. These results align with established literature showing that mitochondrial dysfunction diverts glutamine toward reductive carboxylation to maintain redox homeostasis and supply biosynthetic precursors [13, 56]. This metabolic divergence proposes a model wherein mitochondrial functional status governs LSD1/GLS2’s subtype-specific chemotherapeutic responses in PDAC by directing glutamine metabolic flux.

While GLS is commonly upregulated and acts as oncogenic in cancers, GLS2 expression and its role in tumorigenesis are context-dependent [57, 58]. In hepatocellular carcinoma (HCC) [59], glioblastoma [60], and basal-like breast cancer [61], GLS2 functions as a tumor suppressor. Conversely, it exhibits pro-oncogenic functions in luminal-subtype breast cancer [61] and non-small cell lung cancer (NSCLC) [58]. Recently, Chen et al. revealed that GLS2 promotes immune evasion in PDAC via YAP1 glutamylation, which upregulates PD-L1 expression [62]. Our study now establishes an additional critical role for GLS2 in subtype-specific chemoresistance of PDAC under LSD1 regulation.

In summary, our findings establish LSD1 as a context-dependent epigenetic-metabolic regulator whose therapeutic inhibition requires PDAC subtype stratification. This paradigm is validated in our KPC-derived organoid models, where LSD1 inhibition synergizes with chemotherapy in RSK-subtype organoids but antagonizes treatment in KRAS-subtype organoids. Consequently, RSK-subtype tumors may benefit from combined LSD1 inhibition with chemotherapy, whereas KRAS-subtype tumors might respond better to alternative strategies such as GLS2 inhibition. Future validation in larger human cohorts and patient-derived models will be essential to translate these mechanistic insights into clinically actionable precision therapies for PDAC.

## Methods and materials

### Antibodies and reagents

Mouse monoclonal anti-α-Tubulin (Cell signaling, 3873), rabbit monoclonal anti-LSD1 (Cell signaling, 2139), mouse monoclonal anti-E-cadherin (Santa cruz, 8426), rabbit monoclonal anti-Vimentin (Cell signaling, 5741), rabbit monoclonal anti-GLS (Cell signaling, 49363), mouse monoclonal anti-GLS2 (Invitrogen, MA5-27167), mouse monoclonal anti-GLUL (Biolegend, 856201), rabbit monoclonal anti-PDH (Cell signaling, 3205), rabbit monoclonal anti-LDHA (Cell signaling, 3582), rabbit monoclonal anti-BNIP3 (Cell signaling, 44060), rabbit monoclonal anti-BNIP3L/NIX (Cell signaling, 12396), rabbit monoclonal anti-PINK1 (Cell signaling, 6946), rabbit monoclonal anti-KRAS (Cell signaling, 71835), rabbit monoclonal anti-phospho-DRP1 (Ser616) (Cell signaling, 4494), rabbit monoclonal anti-GAPDH (Cell signaling, 5174), rabbit monoclonal anti-phospho-ERK1/2 (Thr202/Tyr204) (Cell signaling, 4370), rabbit monoclonal anti-ERK1/2 (Cell signaling, 4695), HRP-conjugated secondary antibody (Invitrogen, 31430 and 31460); LSD1 inhibitor ORY-1001 (Sellect, S7795), GLS2 inhibitor C968 (Sigma, SML1327), Complex I inhibitor rotenone (Sigma, 557368); D(+) galactose (Carl roth, 8878), Mtphagy Dye (Dojindo, MT02), Nonyl Acridine Orange (NAO, Biomol, Cay-25545), MitoTracker Deep Red FM (MTDR, Cell signaling, 8778), tetramethylrhodamine ethyl ester (TMRE, Invitrogen, T669), MitoSOX (Invitrogen, M36008) were purchased from the indicated manufacturers. Chemotherapeutic agents, including gemcitabine (Gemcitabin Hexal, Hexal AG) and paclitaxel (NeoTaxan, Hexal AG), were supplied by University Hospital Cologne.

### Cell culture

To represent the heterogeneity of PDAC, cell lines with different KRAS mutation backgrounds were selected. Human pancreatic adenocarcinoma cells L3.6pl (KRAS G12D) were cultured in DMEM (Gibco, USA) supplemented with 10% FBS, 1% vitamin mixture, 1% NEAA, 2mM L-glutamine and 1% penicillin/streptomycin. PDAC patient-derived primary cells TBO368 (KRAS G12R) were cultured in advanced DMEM (Gibco, USA) supplemented with 10% FBS, 2mM L-glutamine and 1% penicillin/streptomycin. HEK293T, MIA PaCa-2 (KRAS G12C), PANC-1 (KRAS G12D), HPAFII (KRAS G12D), and BxPC3 (KRAS wildtype) were cultured in DMEM (Gibco, USA) supplemented with 10% FBS, 2mM L-glutamine and 1% penicillin/streptomycin. All cell lines were tested for mycoplasma contamination. Patient-derived materials were ethically collected from biobank under approval of BIOMASOTA (approved by the Ethics Committee of the University of Cologne, ID: 13-091 and ID:18-274), and informed consent was obtained from all subjects. All methods were performed in accordance with the relevant guidelines and regulations.

### Generation and culture of KPC-derived organoids

KPC (Pdx1-Cre; Kras^G12D/+^;p53^R172H/+^) mouse tumor-derived organoids were kindly provided by Dr. Bo Kong (Heidelberg, Germany) as previously described [63, 64]. Briefly, tumor tissues were minced and digested with collagenase II in Advanced DMEM/F12 for 1–2 h at room temperature. After washing, cell pallets were embedded in Matrigel and seeded in 24-well plates. The complete culture medium consisted of Advanced DMEM/F12 supplemented with 10 mM HEPES, 1× GlutaMAX, 1× B27, 1.25 mM N-acetylcysteine, 10 mM Nicotinamide, 50 ng/mL mEGF, 100 ng/mL hEGF, 10 nM hGastrin I, 100 ng/ml mNoggin, 0.5 µM A83-01, and 10% R-spondin1 condition medium. Organoids were passaged every 7-10 days by digesting in TrypLE Express and re-embedded in Matrigel.

### Quantitative real-time PCR (qRT-PCR)

RNA was extracted from cultured cells or tissue samples with TRI reagent (Sigma-Aldrich). For reverse transcription, High-Capacity cDNA Reverse Transcription Kit (Applied Biosystems, Thermo Fisher Scientific, USA) was used according to the manufacturer’s instructions. Target mRNAs were determined using the Fast SYBR green master mix (Invitrogen) with QuantStudio 7 flex (Applied Biosystems, Thermo Fisher Scientific, USA). Results were repeated 3 times.

### Western blot

Cells were harvested and lysed with RIPA buffer (Cell Signaling, 9806) on ice for 30 min and centrifuged at 12000× g for 10 min. Supernatant was collected, and protein concentration was measured using the BCA method (Thermo Fisher Scientific). Protein samples were boiled in 1x NuPAGE LDS sample buffer (Invitrogen) at 70 °C for 10 min. Twenty microgram protein samples were subjected to 7.5–15% gradient SDS-PAGE gel and transferred to PVDF membrane (MACHEREY-NAGEL, Germany). The membranes were blocked in 1x Roti-Block (Carlroth, Germany) at room temperature for 1 h and were then probed with specific primary antibodies overnight at 4 °C. Blots were incubated with HRP-conjugated secondary antibody (Invitrogen, 31430 and 31460) for 1 h at room temperature. Bands were visualized by SuperSignal West Pico PLUS Chemiluminescent Substrate (Thermo Fisher Scientific, USA) and detected using the ChemoStar ECL Imager (Intas Science Imaging, Germany). Results were repeated 3 times.

### Flow cytometry analysis

For apoptosis analysis, cells were harvested and resuspended in Annexin V binding buffer, with Annexin V (ImmunoTools) and DAPI staining dye, incubated at room temperature for 20 min. For detection of mitochondrial membrane potential, mitochondrial mass and ROS production, cells were stained with TMRE, NAO or MTDR, and MitoSOX at 37°C for 20 min, respectively. All samples were analyzed immediately using a Beckman Coulter CytoFLEX flow cytometer. Results were repeated 3 times. Data were analyzed with Flowjo software (Tree star, Ashland, USA).

### Plasmid constructs

For exogenous expression of LSD1, plasmids pSIN-flag-LSD1-puro and pSIN-empty-puro were used, as described previously [65]. For exogenous expression of BNIP3, KRAS-wildtype, and KRAS-G12D, plasmids were purchased from GenScript (Leiden, The Netherlands). For RNA interference, shRNA sequences for LSD1, BNIP3, and GLS2 were synthesized (Thermo Fisher Scientific) and inserted via AgeI and EcoRI into the Tet-pLKO-puro vector (Addgene, 21915). shRNA target sequences: non-target control, 5’-AGGTAGTGTAATCGCCTTGTT-3’; LSD1, 5’-AGGAAGGCTCTTCTAGCAATA-3’; BNIP3, 5’-GCCACGTCACTTGTGTTTATT-3’; GLS2, 5’-GCCATGGATATGGAACAGAAA-3’. All expression vectors were confirmed by DNA sequencing.

### Lentiviral transduction

Cells stably expressing the shRNA sequence were created by lentiviral transduction. Briefly, HEK293T cells were co-transfected with the transfer vector and packaging vectors (Addgene) using PEI (Sigma-Aldrich) in a mass ratio of 1:3 of DNA/PEI. Medium was changed 24 h later, and virus-containing supernatant was collected and filtered at 48 h and 72 h. Virus-containing supernatant was mixed 1:1 with fresh medium, supplemented with 8 µg/ml polybrene, and used to transduce cells. Puromycin was added 48–72 h later, and selective medium was changed every 2 days and maintained for 1 week. shRNA expression was induced with 1 µg/ml Doxycycline (Sigma-Aldrich).

### Chromatin immunoprecipitation

For chromatin immunoprecipitation, cells were fixed with 1% formaldehyde at room temperature for 12 min and neutralized with glycine (125 mM) for 3 min. Cells were washed with ice-cold PBS for 2 times, then scraped and pelleted. Cell pellets were resuspended in pre-lysis buffer (20 mM Tris, 150 mM NaCl, 1 mM EDTA, 1% Triton X-100, 0.1% SDS, pH 7.5) to eliminate cytosol protein contamination. Cells were pelleted again, resuspended in SDS lysis buffer (50 mM Tris, 10 mM EDTA, 1% SDS, pH 8). Chromatin was sheared and diluted in ChIP dilution buffer (20 mM Tris-HCl, 150 mM NaCl, 1% Triton X-100, 1 mM EDTA, pH 7.5). Then, chromatin samples were subjected to anti-p65 (Cell Signaling, 8252) or normal IgG control (Cell Signaling, 2729) at 4 °C overnight. Then, protein-antibody complexes were precipitated with protein A-Dynabeads (Invitrogen). Immunoprecipitated complexes were washed and eluted with buffer (1% SDS, 0.1 M NaHCO_3_) and then incubated with Proteinase K for 4 h at 65 °C on a thermomixer. DNA was purified using the PCR Clean Up Kit (MACHEREY-NAGEL, Germany) and subjected to quantitative PCR for GLS2 promoter detection. Results were repeated 3 times. Used primers were: GLS2-promoter-for, 5’-GGTGACCCCAGCCTCCT-3’; GLS2-promoter-rev, 5’-TTCTCCGTCTTCCAGGTCCA-3’.

### Stable isotope tracing analysis

The cells were cultured to 70–90% confluence in 6-well plate, then the medium was changed to DMEM without glutamine supplemented with 4 mM U-^13^C-glutamine (Cambridge Isotope Laboratories, CLM-1822-H) and 10% dialyzed FBS (Gibco, 26400044) for additional 24 h. Mass isotopomers of TCA cycle intermediates were measured by IC-MS and LC-MS. Five replicates for each group.

### Seahorse XF Mito Stress Test

OCR and ECAR were determined with Seahorse XF Cell Mito Stress Test Kit (Agilent Technology, #103015-100) according to the manufacturer’s instructions. Briefly, cells were seeded in XF96 cell culture microplates at a density of 20,000 – 30,000 cells/well and cultured for 24 h to reach 90% confluence. One hour before measurement, growth medium was replaced with Seahorse XF Base Medium supplemented with 10 mM glucose, 1 mM pyruvate, and 2 mM L-glutamine (pH 7.4), and cells were incubated at 37 °C in a non-CO₂ incubator. Inhibitors were injected sequentially with oligomycin, FCCP, and rotenone/antimycin A. OCR and ECAR values were normalized to total protein content per well, measured by BCA assay. Six replicates for each group.

### Mitophagy assay

Cells were stained with 100 nM Mtphagy Dye (Dojindo) and 50 nM Lyso Dye (Dojindo) in complete medium at 37 °C for 30 min, followed by three washes with HBSS and cultured in complete medium for an additional 24 hours. Mtphagy Dye accumulates in intact mitochondria and exhibits a weak fluorescence. When Mitophagy is induced, the damaged mitochondria fuse with the lysosome, and then Mtphagy Dye emits a bright fluorescence. All images were acquired using a Leica gSTED super-resolution confocal microscope with a 63× water immersion objective, maintaining cells in HBSS at 37°C during acquisition. Mitophagy was quantified by measuring the intensity of bright Mtphagy fluorescence per cell. Six random cells were selected for each group.

### Public data analysis and survival analysis

Pre-processed TCGA-PAAD and CPTAC RNA-seq data and matched clinical data were downloaded from cBioportal; Peng’s scRNA-seq data were downloaded from China National Center for Bioinformation (CNCB) (Accession: PRJCA001063); Daemen’s metabolomics data were extracted from the paper [8]. All bioinformatic analyses were performed using R (version 4.1.2). scRNA-seq data analyses were conducted using the Seurat package (version 4.1.0) and the monocle package (version 2.22.0); GSEA analysis was conducted using the clusterProfiler package (version 4.14.4), and GSVA score was calculated with the GSVA package (version 2.0.5). Survival analysis was performed using the Kaplan-Meier method, and the difference was tested with the log-rank test. *p* value < 0.05 was considered statistically significant.

### Statistical analyses

Statistical analysis was done using GraphPad Prism 9. Data were presented as mean ± SEM. Statistical comparisons between groups were conducted using unpaired or paired Student’s *t* test (two-sided), or one-way ANOVA as appropriate for the specific experimental design and comparison being made. *p < 0.05, **p < 0.01, ***p < 0.001, ****p < 0.0001, ns: non-significant, *p* > 0.05.

## Supplementary information

Original Blots

Supplementary

## Data Availability

All data generated or analysed during this study are included in this published article and its supplementary information files.
