# Supplementary material for: LSD1-GLS2 axis drives subtype-specific chemoresistance in pancreatic cancer through glutaminolysis reprogramming
Source: Cell Death Dis. 2026 Jul 20;17(1):649. doi: 10.1038/s41419-026-09075-4 (PMC13385623; doi:10.1038/s41419-026-09075-4)

Figure 1G

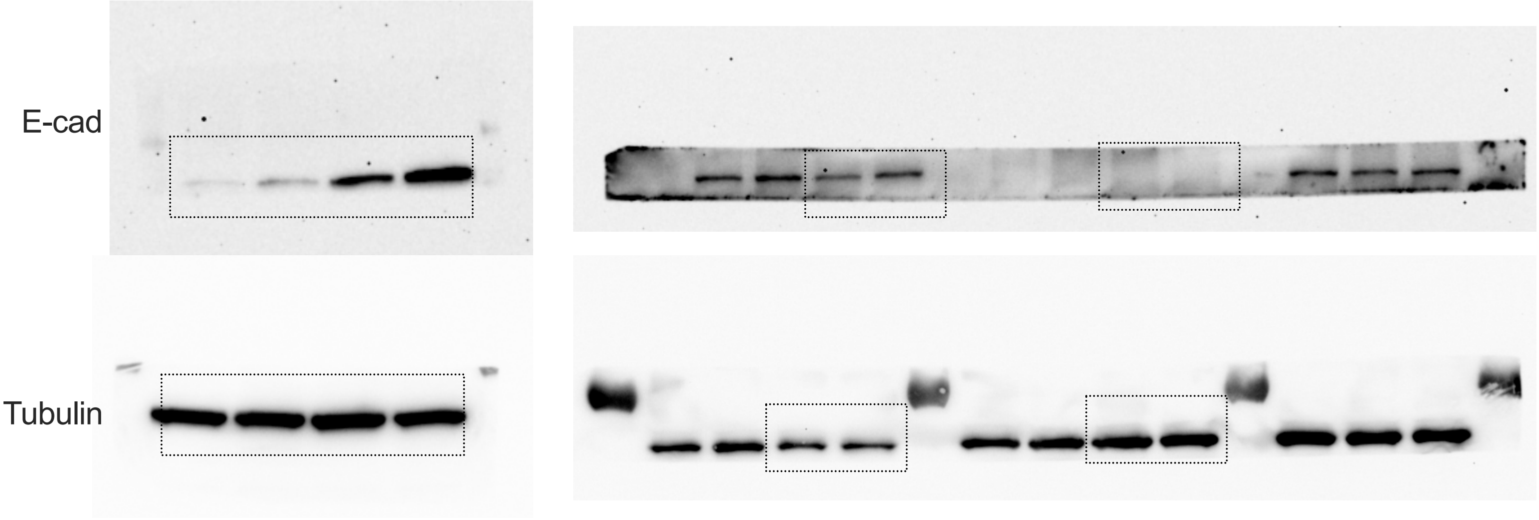

Figure 2J

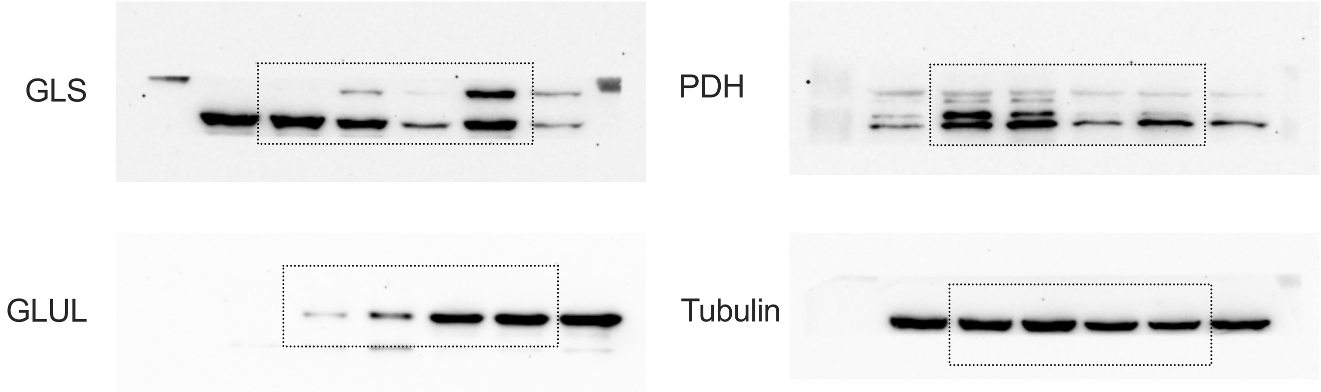

Figure 4I

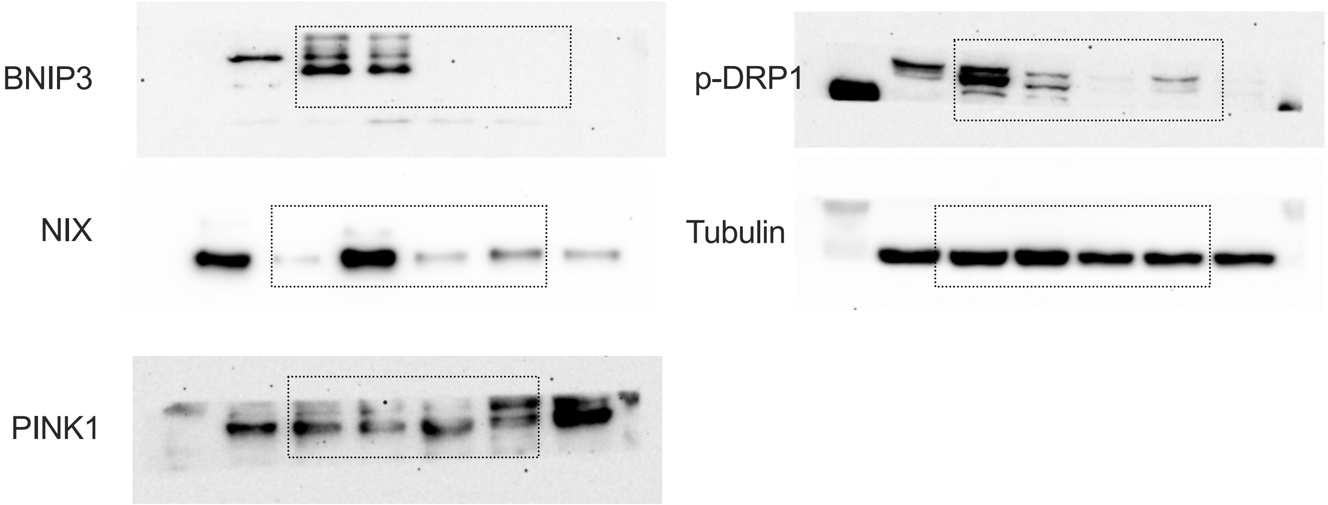

Figure 6A

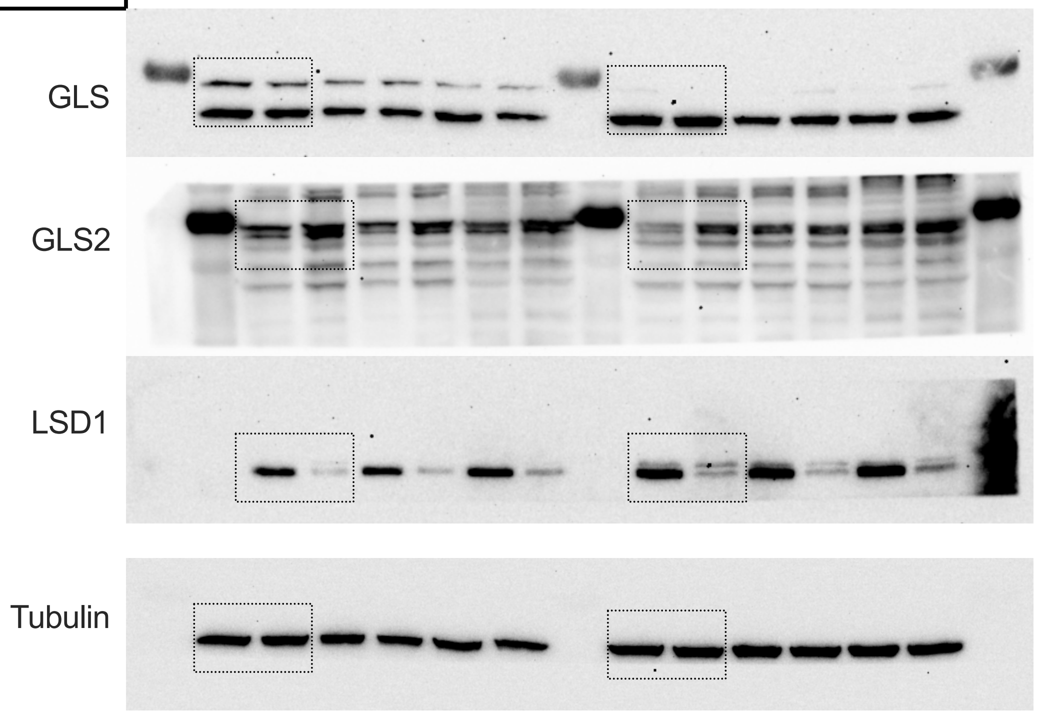

Figure 7C

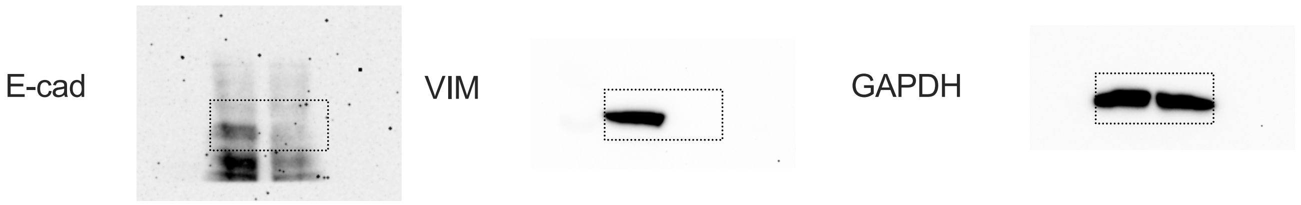

Figure S1E

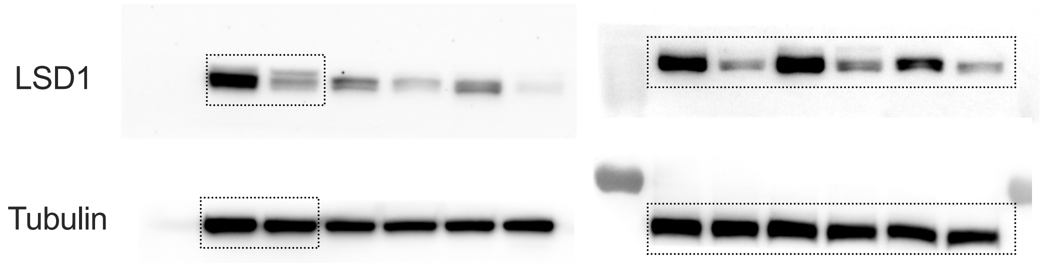

Figure S1F

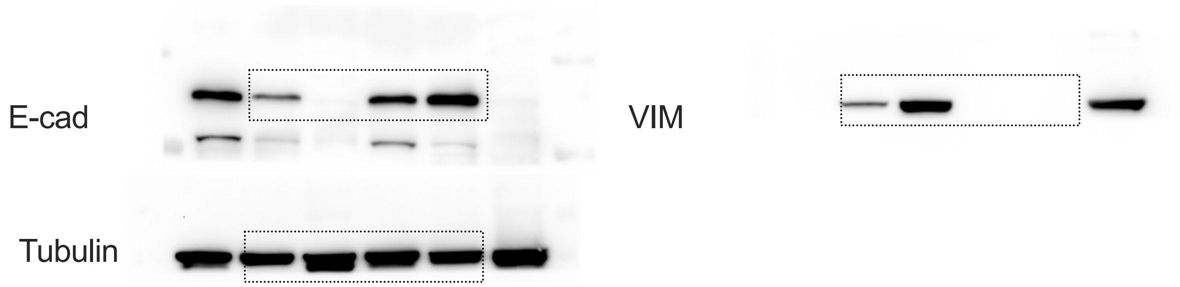

Figure S2A

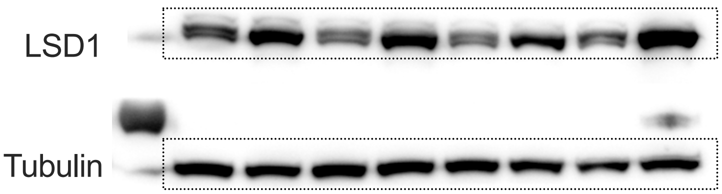

Figure S3G

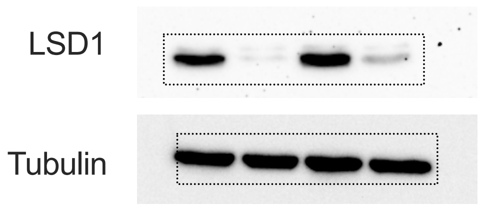

Figure S5A

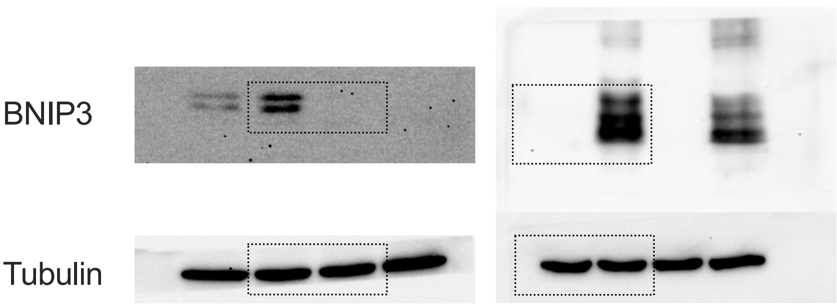

Figure S5D

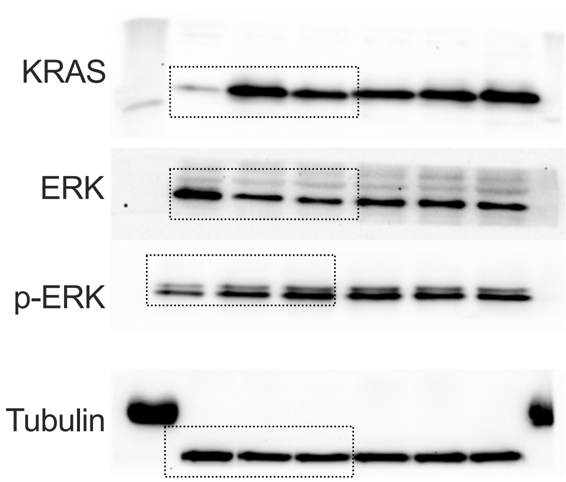

Figure S5E

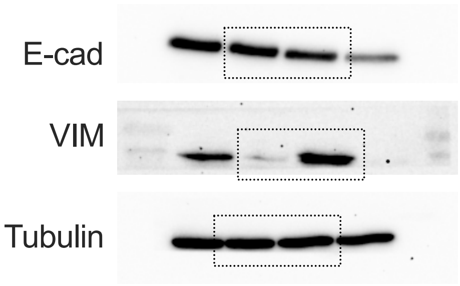

Figure S6A

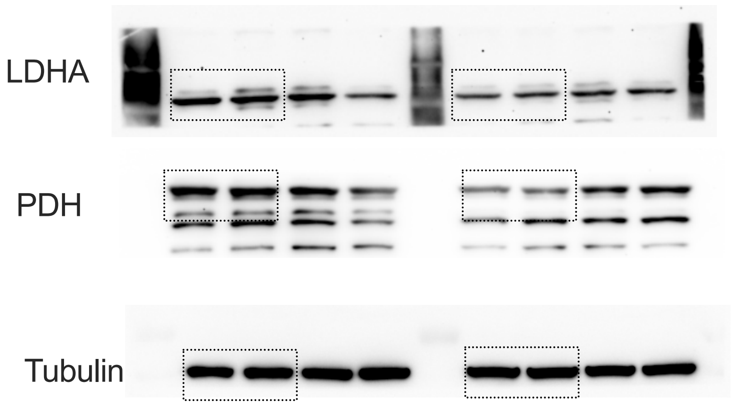

Figure S6D

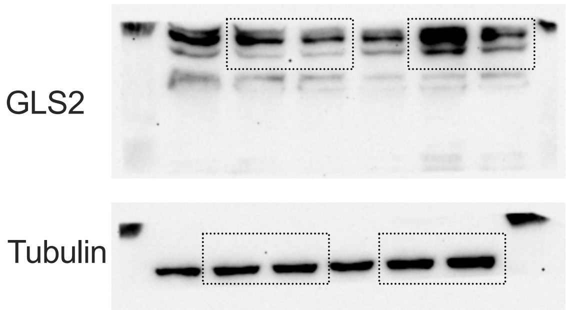

Supplement: Supplementary file 1 — Original Blots [file 41419_2026_9075_MOESM1_ESM.pdf]
