# Supplementary material for: LSD1-GLS2 axis drives subtype-specific chemoresistance in pancreatic cancer through glutaminolysis reprogramming
Source: Cell Death Dis. 2026 Jul 20;17(1):649. doi: 10.1038/s41419-026-09075-4 (PMC13385623; doi:10.1038/s41419-026-09075-4)
Supplement: Supplementary file 2 — Supplementary [file 41419_2026_9075_MOESM2_ESM.pdf]

Supplementary Data

The Supplementary Data consist of:

Supplementary Methods

Supplementary Figures S1-6

Supplementary Methods

Metabolomics analysis

Levels of intermediates (organic acids, sugar phosphates) of the glycolysis and the citric acid cycle in cells were determined by Anion-Exchange Chromatography coupled to Electrospray Ionization High-Resolution Mass Spectrometry (IC-ESI-HRMS) using a procedure previously described method with several modifications: Cells from a 10 cm culture dish per were suspended in 600 µl of ice-cold methanol/acetonitrile/water 5:3:2 (v/v/v) using the Precellys 24 Homogenisator (Peqlab) at 6,400 rpm for 10 sec twice with a 5-sec pause. To 300 µl of homogenate, 25 µl of a mixture of isotope-labeled internal standards in Milli-Q water (50 µM $^{13}C_6$-D-glucose-6-phosphate ($^{13}C_6$-G6P) and 50 µM D_4-succinic acid (D_4-SUC), both Eurisotop) were added. The samples were shaken in a ThermoMixer C (Eppendorf) at 4 °C and 900 rpm for 20 min. After centrifugation (16,100 RCF, 20 min, 4 °C), the clear supernatants were dried under a stream of nitrogen. The residues were resolved in 100 µl of Milli-Q water and centrifuged (16,100 RCF, 20 min, 4 °C) again. 80 µl of the supernatants were transferred to autoinjector vials and immediately measured. IC-HRMS analysis was performed using a Dionex Integrion RFIC system (Thermo Scientific) equipped with a Dionex IonPac AS11-HC column (2 mm × 250 mm, 4 µm particle size, Thermo Scientific) and a Dionex IonPac AG11-HC guard column (2 mm × 50 mm, 4 µm, Thermo Scientific) and coupled to a Q Exactive HF quadrupole-orbitrap mass spectrometer (Thermo Scientific). 5 µl of sample were injected using a Dionex AS-AP at 5 °C. The IC was operated at a flow rate of 0.38 ml/min with a potassium hydroxide gradient which was produced by an eluent generator with a potassium hydroxide cartridge and Milli-Q water. The gradient started with 10 mM KOH over 3 min, 10−50 mM from 3 to 12 min, 50−100 mM from 12 to 19 min, held at 100 mM from 19 to 25 min, and re-equilibrated at 10 mM for 3 min. The total run time was 28 min. An Dionex ADRS 600, 2 mm suppressor was operated with 95 mA, and methanol was used to produce a make-up flow at a flow rate of 0.15 ml/min. The mass spectrometer was operated in the negative ion mode. Full

MS scans in the range of m/z 60-900 were acquired with a resolution of 120,000, an Automatic Gain Control (AGC) target value of 1 x 10^6 and a maximum injection time (IT) of 240 ms. Spectrum data were collected in the centroid mode. The ESI source was operated with flow rates for sheath gas, auxiliary gas, and sweep gas of 50, 14 and 3, respectively. The spray voltage setting was 2.75 kV, the capillary temperature 230 °C, the S-lens RF level 45, and the auxiliary gas heater temperature 380 °C. The exact m/z traces of the internal standards and the endogenous metabolites were extracted and integrated using the TraceFinder 5.1 software (Thermo Scientific). Endogenous metabolites were quantified by normalizing their peak areas to those of the internal standards: $^{13}C_6$-G6P was used for sugar phosphates and D_4-SUC for organic acids. The peak area ratios were normalized to the protein content, which was determined in an additional cell pellet replicate per sample group.

Transmission electron microscopy

Cells were grown on small discs of aclar foil (Science Services, #E50425-10) and fixed for 1 h in 2% Glutaraldehyde (Sigma, # G5882-100ML) with 2.5 % Sucrose (Roth, # 4621.1) and 3mM $CaCL_2$ (Sigma, # C7902-500G) in 0.1M HEPES buffer (Sigma, # C7902-500G) pH 7,4. Samples were washed three times with 0.1M HEPES buffer and incubated with 1% Osmiumtetroxid (Science Services, # E19190) and 1% Potassium hexacyanoferrat (Sigma, # P8131) for 1 h at 4°C. After 3x5min wash with 0.1M Cacodylate buffer (Applichem, # A2140,0100), samples were dehydrated at 4°C using ascending ethanol series (50%, 70%, 90%, 3x100%) for 7 min each. Infiltration was performed with a mixture of 50% Epon/ethanol for 1h, 70% Epon/ethanol for 2h and with pure Epon (Science Services, # E14120) overnight at 4°C. Samples were embedded into TAAB capsules (Agar Scientific, #G3744) and cured for 48 h at 60°C. Ultrathin sections of 70 nm were cut using an ultramicrotome (Leica Microsystems, UC6) and a diamond knife (Diatome, Biel, Switzerland). Sections were stained with 1.5 % uranyl acetate (Agar Scientific, # R1260A) for 15 min at 37°C and with 3 % Reynolds

lead citrate solution made from Lead (II) nitrate (Roth, # HN32.1) and tri-Sodium citrate dehydrate (Roth, # 4088.3) for 4 min. Images were acquired using a JEM-2100 Plus Transmission Electron Microscope (JEOL) operating at 80kV equipped with a OneView 4K camera (Gatan).

Supplementary figures

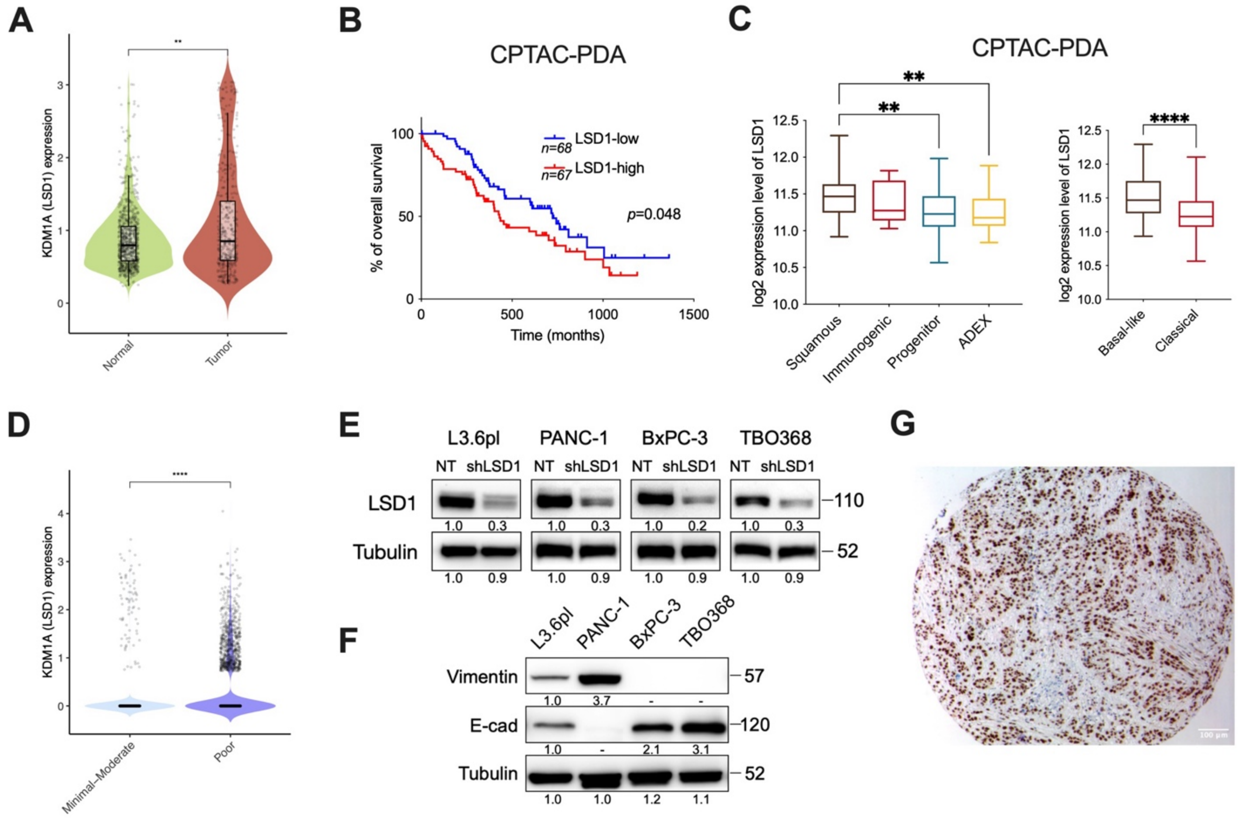

Figure S1. LSD1 is a prognostic marker in PDAC regulating proliferation and E-cadherin repression. A) LSD1 expression analysis in PDAC using single-cell RNA-sequencing data from GSE212966. LSD1 expression was elevated in tumor cells as compared to normal epithelial cells. Wilcoxon rank-sum test. B-C) CPTAC-PDA dataset analysis. B) Kaplan-Meier survival analysis shows high LSD1 expression is associated with poor survival of PDAC patients. Log-rank. C) Differential LSD1 expression across PDAC molecular subtypes, showing significant enrichment in squamous/basal-like tumors. Data are presented as box-and-whisker plot (Min to Max). Group comparisons were performed using unpaired two-tailed Student's *t*-tests. For analysis involving three or more groups, one-way ANOVA with Sidak's correction was applied. D) LSD1 expression analysis in PDAC using single-cell RNA-sequencing data from GSE202051. LSD1 expression was higher in patients with poor response to neoadjuvant treatments as compared to patients with minimal-moderate response.

Wilcoxon rank-sum test. E) Validation of LSD1 knockdown at protein level. F) Western blot analysis of Vimentin and E-cadherin. Tubulin served as loading control. G) Representative immunohistochemistry (IHC) staining of LSD1 in human PDAC. $**p < 0.01$, $****p < 0.0001$.

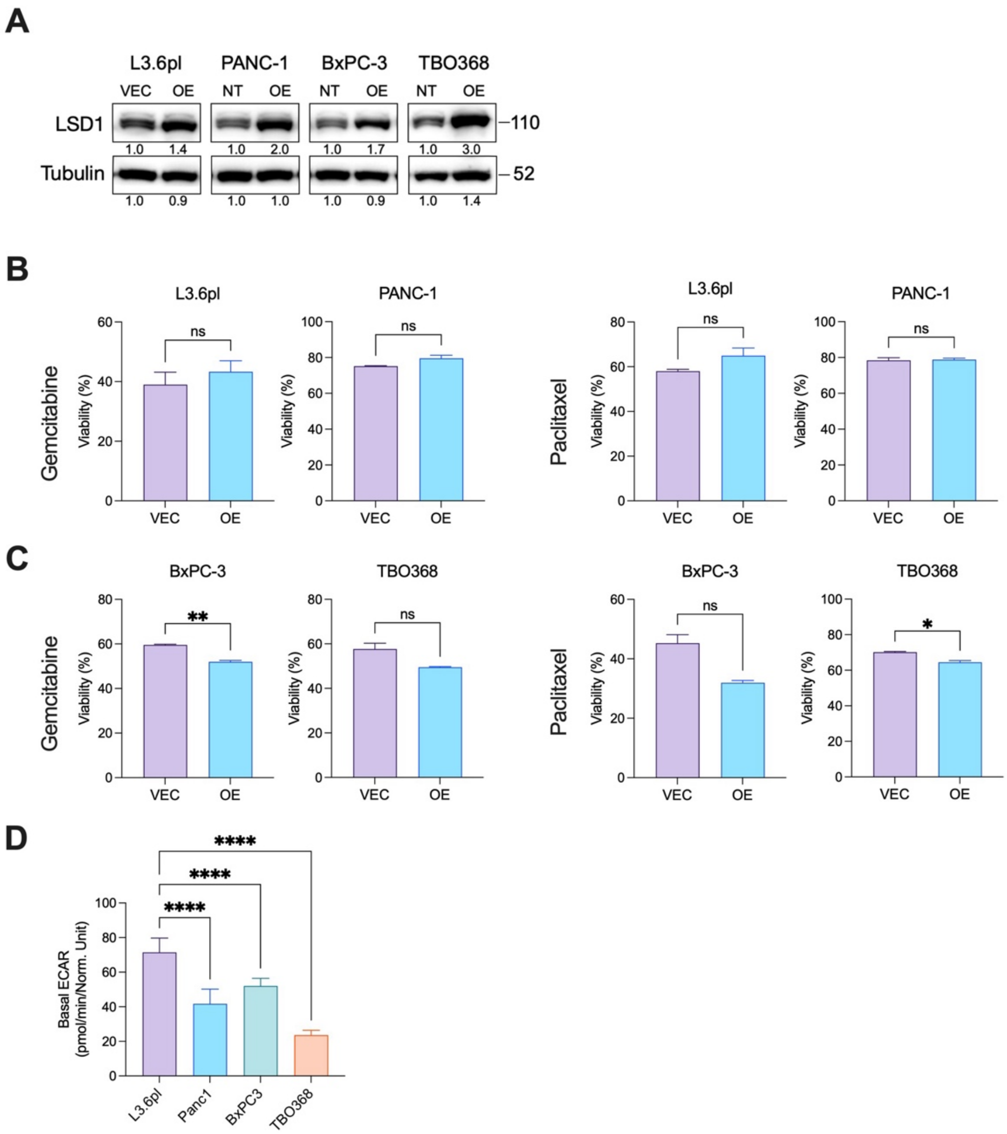

Figure S2. LSD1 exhibits context-dependent roles in PDAC chemotherapeutic response. A) Validation of LSD1 overexpression at protein level. B-C) Chemosensitivity analysis following LSD1 overexpression. Cells were treated with either gemcitabine (3 ng/ml for L3.6pl and 400 ng/ml for others) or paclitaxel (25 nM) for 48-72 hours, then assessed by Annexin V/DAPI apoptosis assay. D) Basal extracellular acidification rate (ECAR) of four PDAC cells were demonstrated as bar chart. One-way ANOVA. Data represent mean ± SEM of three independent

experiments. Statistical significance was determined by paired two-tailed Student's *t*-test otherwise indicated. *$p < 0.05$, **$p < 0.01$, ****$p < 0.0001$, ns: non-significant, $p>0.05$.

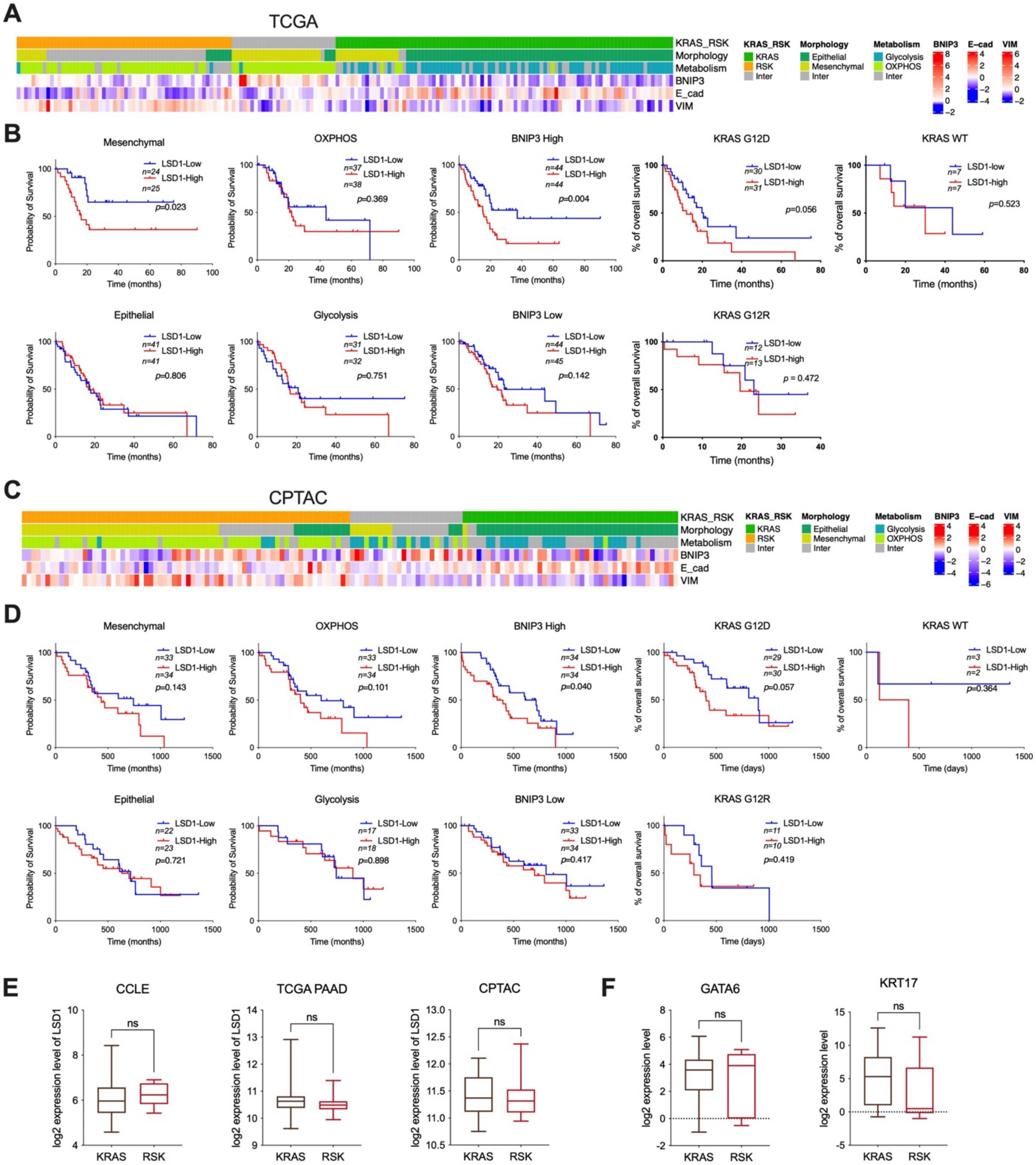

A
TCGA
KRAS_RSK
Morphology
Metabolism
BNIP3
E_cad
VIM
KRAS_RSK
KRAS
RSK
Inter
Morphology
Epithelial
Mesenchymal
Inter
Metabolism
Glycolysis
OXPHOS
Inter
B
Mesenchymal
OXPHOS
BNIP3 High
KRAS G12D
KRAS WT
Epithelial
Glycolysis
BNIP3 Low
KRAS G12R
LSD1-Low
LSD1-High
Probability of Survival
% of overall survival
Time (months)
p=0.023
p=0.369
p=0.004
p=0.056
p=0.523
p=0.806
p=0.751
p=0.142
p = 0.472
C
CPTAC
D
Time (days)
p=0.143
p=0.101
p=0.040
p=0.057
p=0.364
p=0.721
p=0.898
p=0.417
p=0.419
E
CCLE
TCGA PAAD
CPTAC
log2 expression level of LSD1
ns
KRAS
RSK
F
GATA6
KRT17
log2 expression level

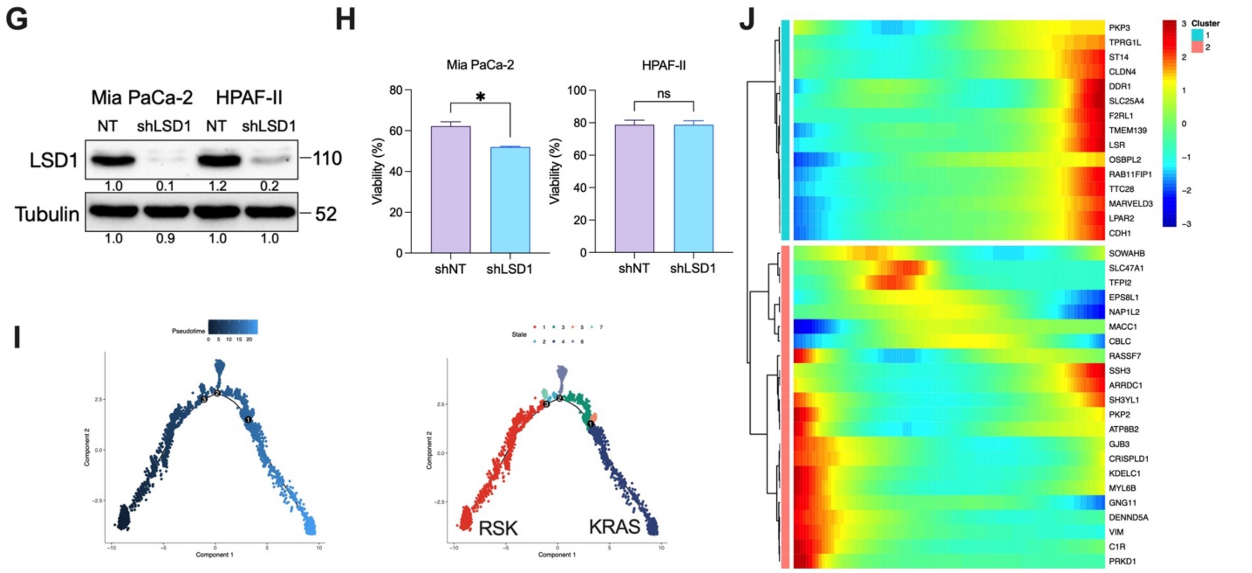

Figure S3. KRAS-RSK subtyping determines the context-dependent roles of LSD1 in PDAC. A-B) TCGA-PAAD dataset analysis. A) Subtype stratification of PDAC tumor samples. Unsupervised clustering of 177 tumor samples using KRAS_RSK_sig, Morphology_sig, and Metabolism_sig. E-cadherin, Vimentin, and BNIP3 expression are annotated. B) Kaplan-Meier survival analysis stratified by molecular subtypes, BNIP3 expression level, and KRAS mutation status. Log-rank. C-D) CPTAC-PDA dataset analysis. C) Subtype stratification of PDAC tumor samples. Unsupervised clustering of 140 tumor samples using KRAS_RSK_sig, Morphology_sig, and Metabolism_sig. E-cadherin, Vimentin, and BNIP3 expression are annotated. D) Kaplan-Meier survival analysis stratified by molecular subtypes, BNIP3 expression level, and KRAS mutation status. Log-rank. E) LSD1 expression across KRAS-RSK subtypes in CCLE cell lines, TCGA-PAAD cohort, and CPTAC-PDA cohort. F) Expression of canonical classical and basal-like markers (GATA6 and KRT17) across KRAS-RSK subtypes in CCLE cell lines. G) Validation of LSD1 knockdown in Mia PaCa-2 and HPAF-II. H) Chemosensitivity analysis following LSD1 knockdown. Cells were treated with gemcitabine (400 ng/ml) for 72 hours, then assessed by Annexin V/DAPI apoptosis assay. I) Trajectory analysis of Peng's scRNA-seq data. J) Pseudotime heatmap demonstrated that KRAS and RSK subtype markers localized to opposite ends of the

pseudotime continuum. Data represent mean ± SEM of three independent experiments. Statistical significance was determined by unpaired two-tailed Student's t-test otherwise indicated. *$p < 0.05$, ns: non-significant, $p>0.05$.

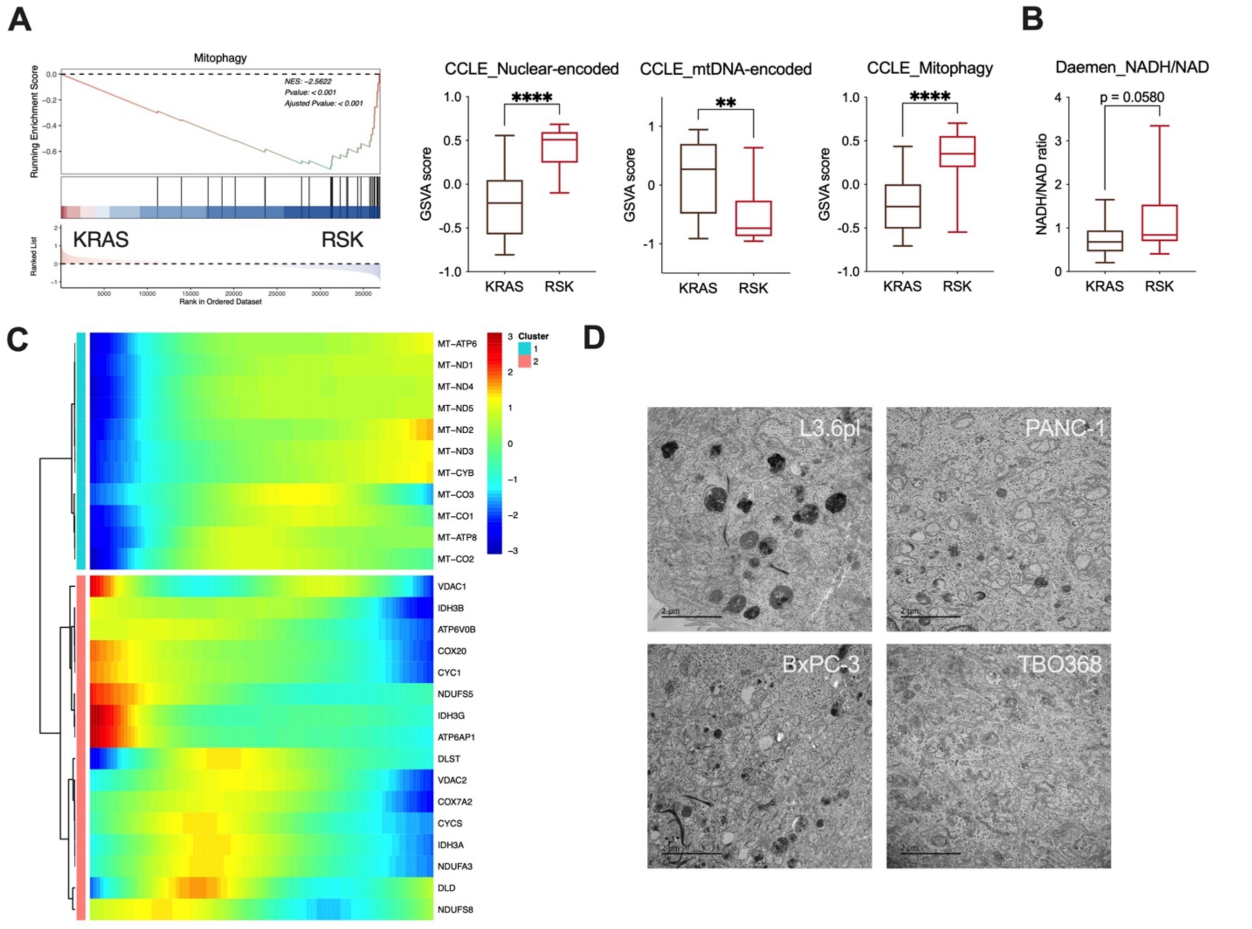

Figure S4. Mitochondrial dysfunction and mitophagy deficiency serve as hallmarks that distinguish KRAS-RSK subtypes of PDAC**.** A) CCLE dataset analysis. GSEA demonstrated significant enrichment of Mitophagy signature in RSK subtype. GSVA demonstrated significantly elevated Nuclear-encoded mitochondrial gene score and Mitophagy score in RSK subtype, while mtDNA-encoded mitochondrial gene score was elevated in KRAS subtype. B) Metabolomic data from Daemen et al. shows higher NADH/NAD+ ratio in RSK subtype, though not statistically significant. C) Trajectory analysis of Peng's scRNA-seq data. Pseudotime heatmap demonstrated that nuclear-encoded and mtDNA-encoded mitochondrial genes were localized to opposite ends of the pseudotime continuum, corresponding to RSK and KRAS subtypes, respectively. D) Transmission electron microscopy (TEM) revealed mitochondria with crowed and disorganized ultrastructure in BxPC-3 and TBO368 compared to L3.6pl and PANC-1. Statistical significance was determined by

unpaired two-tailed Student's *t*-test otherwise indicated. $^{**}p < 0.01$, $^{****}p < 0.0001$, ns: non-significant, $p>0.05$.

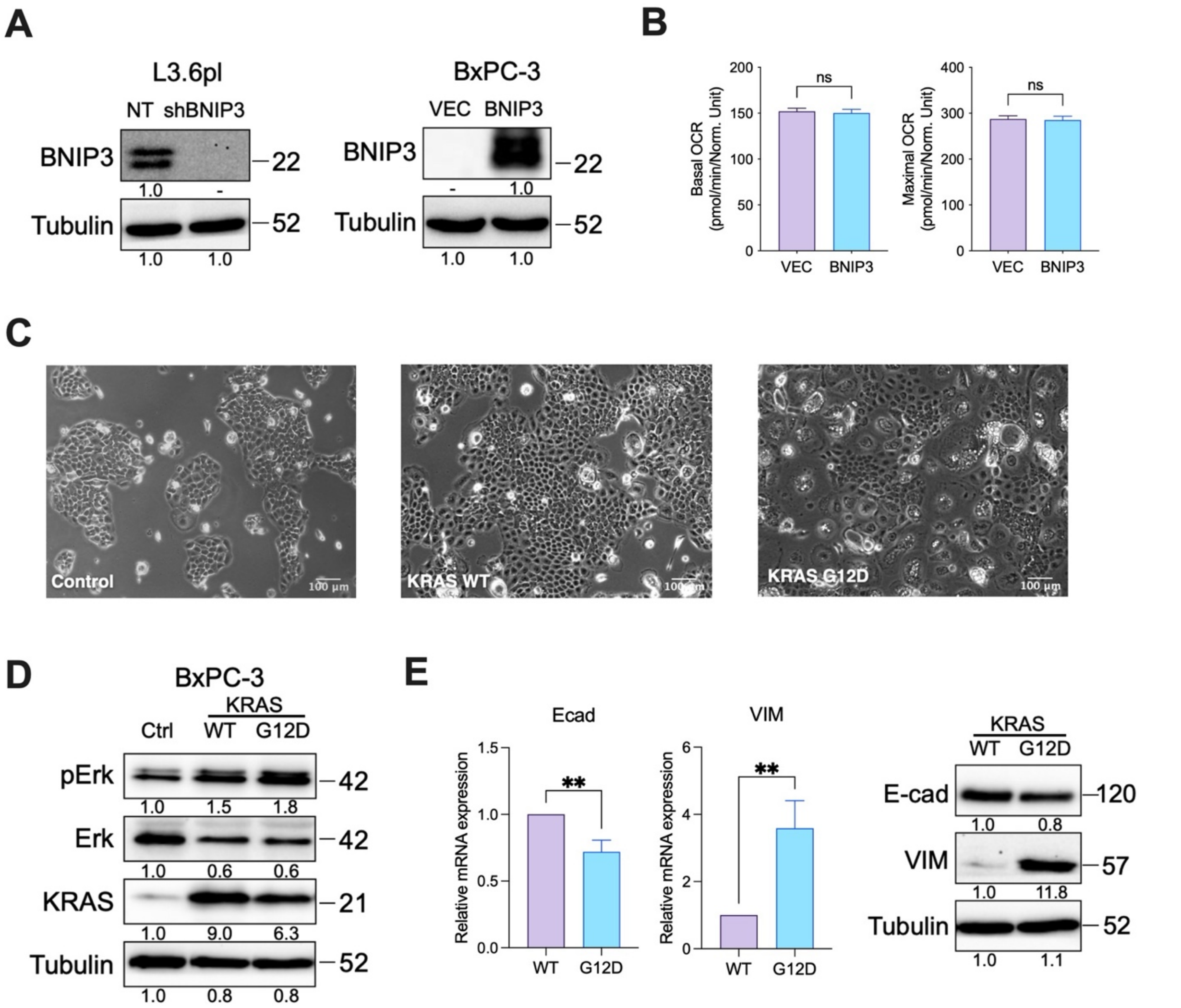

Figure S5. Mitochondrial targeting overrides LSD1's context-dependent regulation of chemotherapeutic response in PDAC subtypes. A) Validation of BNIP3 modulation at protein level. B) Mitochondrial respiration profiling of BxPC3 cells following exogenous BNIP3 expression. C-D) Engineered KRAS expression in BxPC-3. C) Brightfield imaging reveals morphological changes induced by KRAS-G12D. D) Western blot confirms exogenous expression of KRAS-wildtype (KRAS-WT) and KRAS-G12D constructs. E) KRAS-G12D drives EMT reprogramming. KRAS-G12D expression significantly upregulated Vimentin and downregulated E-cadherin at both mRNA and

protein levels, indicating a mesenchymal phenotypic shift. Statistical significance was determined by paired two-tailed Student's *t*-test. **$p < 0.01$, ****$p < 0.0001$, ns: non-significant, $p>0.05$.

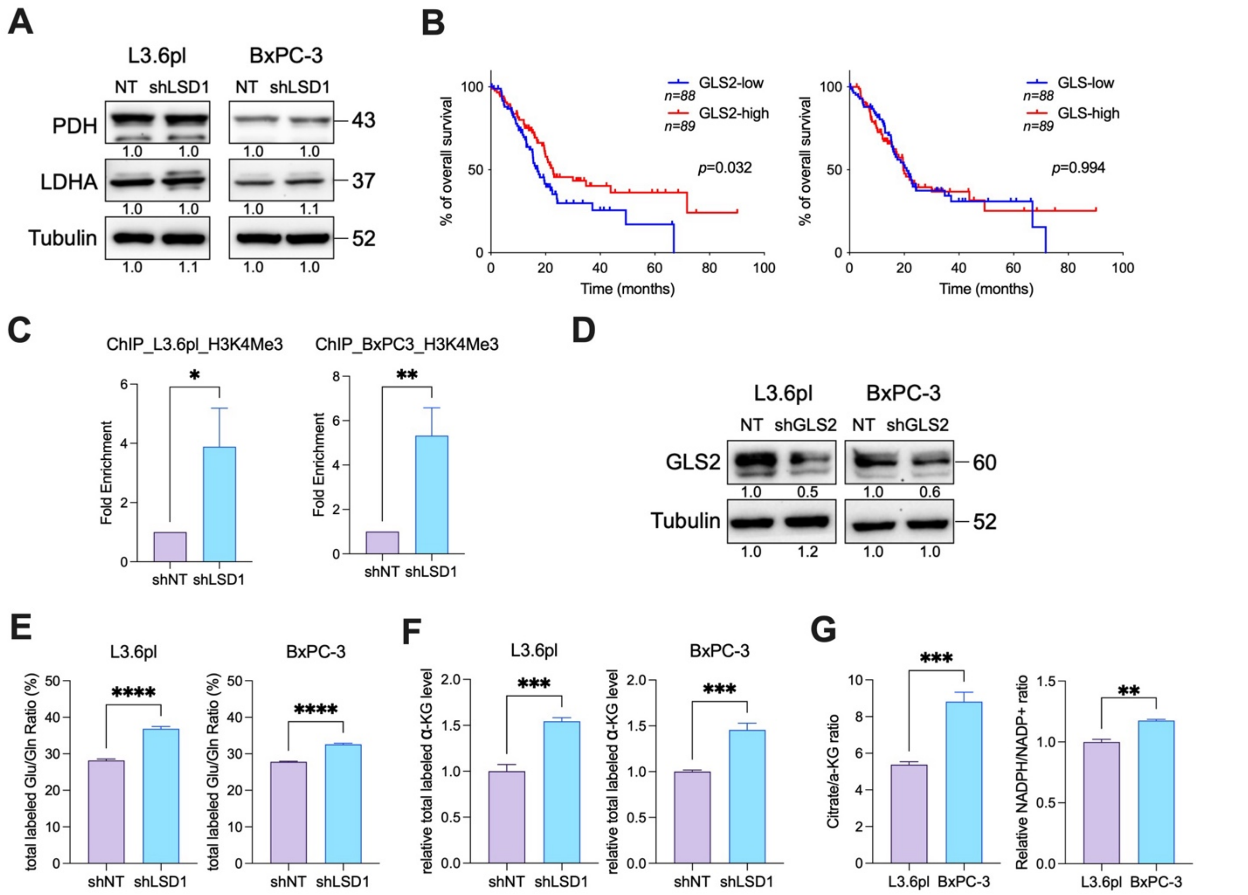

Figure S6. LSD1 shapes chemotherapeutic response in PDAC through GLS2-mediated glutamine metabolic reprogramming. A) Western blot analysis of PDH and LDHA. Tubulin served as loading control. B) Kaplan-Meier survival analysis revealed that high GLS2 expression predicts improved overall survival in PDAC patients, contrasting with no significant association for GLS expression. Log-rank. C) ChIP assay revealed increased H3K4me3 enrichment at GLS2 promoter following LSD1 knockdown. Paired *t*-test. D) Validation of GLS2 knockdown at protein level. E) Total labeled glutamate/glutamine ratio was increased after LSD1 knockdown. F) Total labeled α-KG level was elevated after LSD1 knockdown. G) Citrate/a-KG ratio and NADPH/NADP+ ratio were both elevated in BxPC-3 relative to L3.6pl. Statistical significance was determined by unpaired two-tailed Student's *t*-test otherwise indicated. $^{**}p < 0.01$, $^{***}p < 0.001$, $^{****}p < 0.0001$.
